# Daughter Cell Identity Emerges from the Interplay of Cdc42, Septins, and Exocytosis

**DOI:** 10.1016/j.devcel.2013.06.015

**Published:** 2013-07-29

**Authors:** Satoshi Okada, Marcin Leda, Julia Hanna, Natasha S. Savage, Erfei Bi, Andrew B. Goryachev

**Affiliations:** 1 Department of Cell and Developmental Biology, Perelman School of Medicine, University of Pennsylvania, Philadelphia, PA 19104, USA; 2 SynthSys—Centre for Systems and Synthetic Biology and Institute for Cell Biology, School of Biological Sciences, University of Edinburgh, Edinburgh EH9 3JR, UK

## Abstract

Asymmetric cell division plays a crucial role in cell differentiation, unequal replicative senescence, and stem cell maintenance. In budding yeast, the identities of mother and daughter cells begin to diverge at bud emergence when distinct plasma-membrane domains are formed and separated by a septin ring. However, the mechanisms underlying this transformation remain unknown. Here, we show that septins recruited to the site of polarization by Cdc42-GTP inhibit Cdc42 activity in a negative feedback loop, and this inhibition depends on Cdc42 GTPase-activating proteins. Combining live-cell imaging and computational modeling, we demonstrate that the septin ring is sculpted by polarized exocytosis, which creates a hole in the accumulating septin density and relieves the inhibition of Cdc42. The nascent ring generates a sharp boundary that confines the Cdc42 activity and exocytosis strictly to its enclosure and thus clearly delineates the daughter cell identity. Our findings define a fundamental mechanism underlying eukaryotic cell fate differentiation.

## Highlights

• Septins provide negative feedback to Cdc42 activity that depends on Cdc42 GAPs
• Septin ring is formed by highly focused polarized exocytosis
• Suppression of exocytosis causes chasing behavior of septins
• Septin ring size is primarily determined by size of the underlying Cdc42 cluster

## Graphical Abstract

**Figure:**
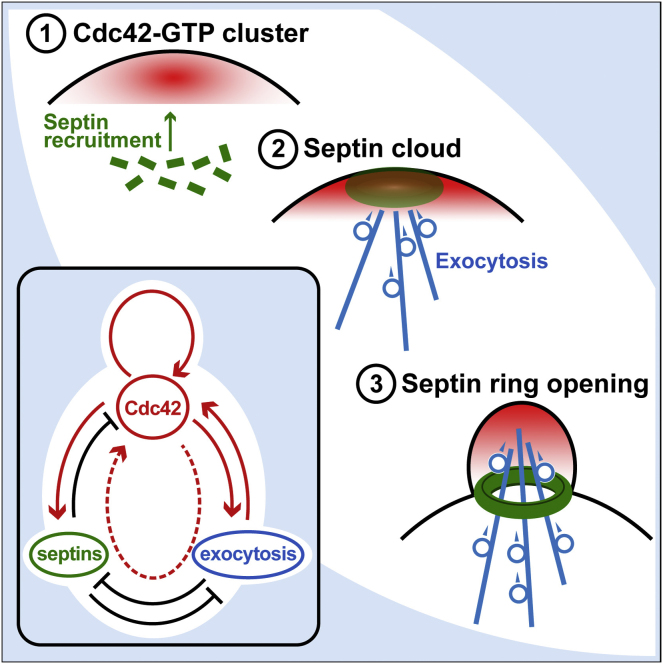

## Teaser

Okada et al. combine live-cell imaging and computational modeling to show that in budding yeast, Cdc42-GTP-dependent septin accumulation at the incipient bud site is sculpted into a ring structure by highly focused exocytosis. Translocation of Cdc42-GTP and its effectors within the septin ring underlies delineation of daughter cell identity.

## Introduction

Asymmetric cell division plays a key role in differential inheritance of cell fate determinants and segregation of damaged material to one daughter cell (Macara and Mili, 2008). Budding yeast, in which mother and daughter possess distinct developmental programs and replicative life spans, provides an important model system to study molecular mechanisms of cell fate differentiation. To prevent intermixing of material between cells sharing contiguous membranes, diffusion barriers, recently associated with septin cytoskeleton (Caudron and Barral, 2009), are required. Septins are conserved GTP-binding proteins that form hetero-oligomeric complexes capable of assembling into higher-order structures, such as filaments, rings, and gauzes (Oh and Bi, 2011). They play important roles in diverse cellular functions and human diseases, including polarized cell growth, cytokinesis, mitosis, cell migration, bacterial infection, cancer, and neurodegenerative diseases (Saarikangas and Barral, 2011; Spiliotis and Nelson, 2006). Diffusion barriers based on polymeric septin rings are found not only at the division site of fungal cells but also at the base of primary cilia, dendritic spines, and in the annuli of sperm tails (Caudron and Barral, 2009). However, the mechanisms underlying septin ring biogenesis remain unknown in any system.

In budding yeast, five mitotic septins (Cdc3, Cdc10, Cdc11, Cdc12, and Shs1) form rod-shaped hetero-oligomeric complexes, which polymerize into filaments that further organize into a ring structure at the presumptive bud site (PBS) (McMurray and Thorner, 2009). Upon bud emergence, the septin ring matures into a collar that is localized at the narrow neck between mother and daughter. Recently, using polarized fluorescence microscopy and cryo-electron tomography, much progress has been made in understanding the ultrastructure of the septin ring and collar (Bertin et al., 2012; DeMay et al., 2011; Vrabioiu and Mitchison, 2006). Despite careful description of septin accumulation at the PBS (Chen et al., 2011; Iwase et al., 2006) and the demonstration of the essential role of Cdc42 in this process (Caviston et al., 2003; Iwase et al., 2006; Kadota et al., 2004), the precise molecular mechanisms that control septin ring formation, its position and size, remain poorly understood. Thus, it is still unclear what makes the accumulating septin polymers form a ring (Chen et al., 2011).

Here, we demonstrate that septin ring formation is equivalent to the emergence of a qualitatively distinct membrane domain that is destined to become a daughter cell. We discover a crucial role of exocytosis in shaping septins into a ring and demonstrate that interlinked feedback loops between Cdc42 activity, septins, and exocytosis are essential for this process. We develop a whole-cell systems biology model that integrates the formation of the Cdc42 cluster, recruitment of septins, and exocytosis to explain the simultaneous emergence of the septin ring and the nascent bud as a single morphogenetic event that differentiates daughter from mother long before cytokinesis separates them irreversibly.

## Results

### Cdc42-GTP Drives Accumulation of Septins that May Locally Suppress Cdc42 Activity in a Negative Feedback Loop

Previously, we have shown that Cdc42-GTP is required for septin recruitment to the PBS (Iwase et al., 2006). To explore further how Cdc42 might control septin ring assembly, we determined the precise spatiotemporal relationship between Cdc42-GTP (detected by Gic2PBD-RFP; see Supplemental Experimental Procedures, Figure S1, and Movie S1 available online) and septins (Cdc3-GFP) by time-lapse microscopy. Because mother cells enter the next cell cycle immediately after cytokinesis whereas daughter cells have a prolonged G1 phase, the dynamics of Cdc42-GTP and septins was monitored only in mother cells from the time of cytokinesis to a small-budded stage. This allows clear visualization of the entire sequence of polarization events at the PBS.

**Figure 1:**
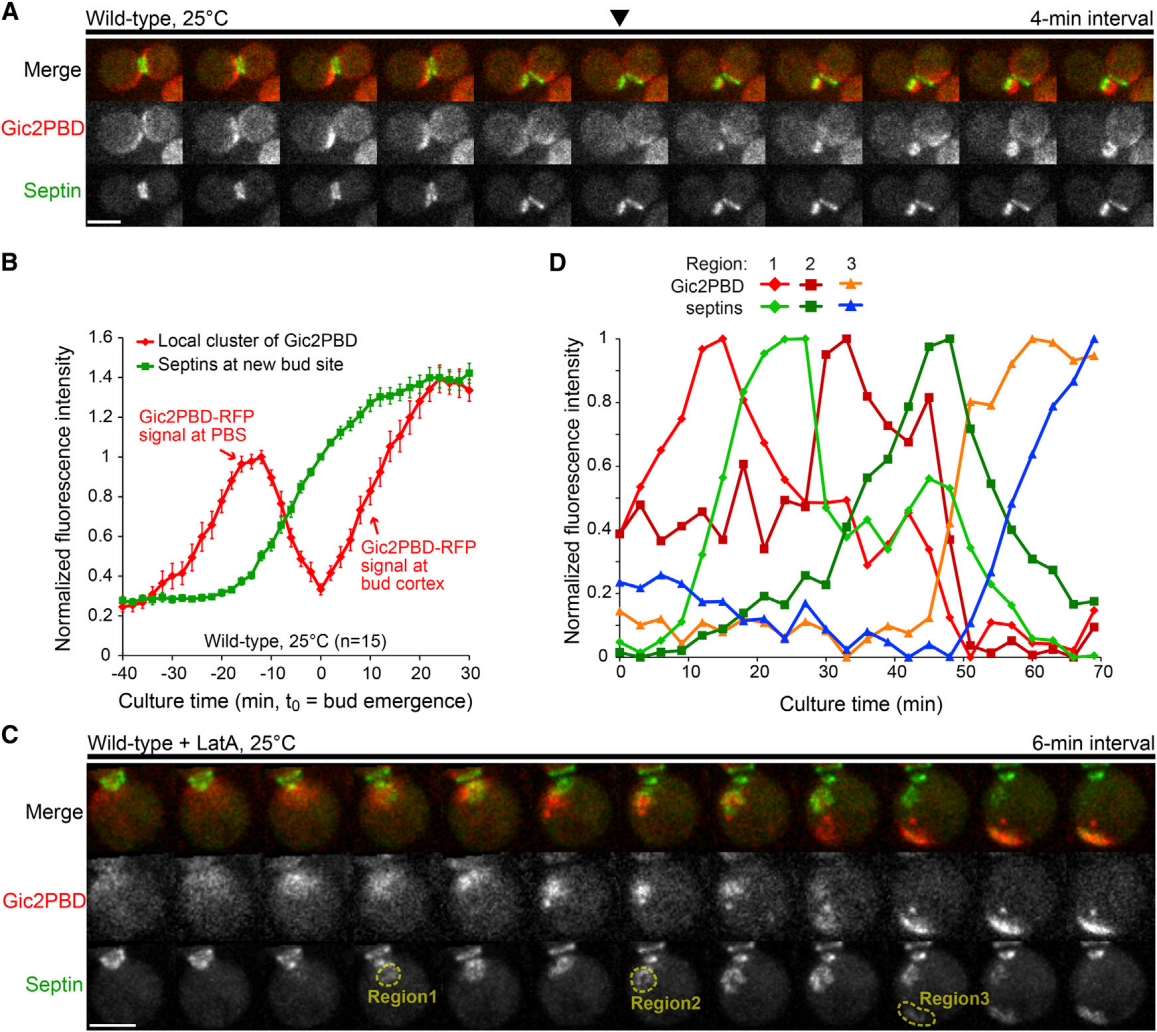
Cdc42-GTP Recruits Septins during Bud Emergence and Chasing (A) Time-lapse analysis of Cdc42-GTP (Gic2PBD-RFP; see also Figure S1) and septin (Cdc3-GFP) dynamics in wild-type cells. Arrowhead indicates bud emergence. See also Movie S1. (B) Average fluorescence intensity of Gic2PBD and Cdc3. Gic2PBD intensity was quantified using a threshold method (see Supplemental Experimental Procedures and Figure S2). Septin intensity was quantified using a fixed region of interest (ROI) set at the PBS. Average intensities are reported after background subtraction and normalization. Error bars represent SEM. (C) Chasing of Cdc42-GTP cluster by septins in a latA-treated cell. See also Movie S2. (D) Quantification of fluorescence intensity of Gic2PBD-RFP and Cdc3-GFP within the ROIs marked on (C). Scale bars represent 3 μm.

Surprisingly, erratic and rapidly changing flashes of Cdc42 activity were observed around the bud neck even before cytokinesis (Figure 1A; Movie S1). Cdc42-GTP signal intensified progressively over time and eventually consolidated as a stable bright cluster that clearly defined the PBS 10–12 min prior to bud emergence. Due to the constantly changing morphology and position of Cdc42 activity, we used a threshold method to quantify the Cdc42-GTP signal overall in the cell and specifically within the PBS cluster after its establishment (see Supplemental Experimental Procedures and Figure S2). Septins began to accumulate at the PBS 14–16 min before bud emergence, approximately concurrent with the maximum of the Gic2PBD-RFP signal (Figure 1B). As septins accumulated further, the Gic2PBD-RFP signal began to decline. Although Cdc42 activity at the PBS continuously decreased, the morphology of the nascent septin structure changed rapidly from an amorphous cloud or cap in the center of the Cdc42 cluster to a ring structure, which occurred simultaneously with bud emergence. Immediately after bud emergence, the intensity of Gic2PBD-RFP signal surged up, but only within the bud cortex separated from mother by the septin ring. Qualitatively similar, albeit less pronounced, changes in the total Cdc42 were observed at the PBS using GFP-Cdc42 (data not shown). The transformation of amorphous septin accumulation into a ring took 2–3 min, a time frame similar to the interval of our imaging, thus leaving the finer details of this transition unresolved.

To better characterize this critical transition, we performed imaging in the presence of 100 μM latrunculin A (latA), which at this concentration disrupts both actin cables and patches, as well as delays septin ring formation (Iwase et al., 2006; Kadota et al., 2004). Indeed, in contrast with 96% of control cells, only ∼50% of the latA-treated cells formed a new septin ring during the time of imaging (see more discussion on the transitional structures of septins in latA-treated cells below). A pronounced change in membrane curvature within the septin ring opening signaled the emergence of tiny hemispherical protrusions that accompanied formation of the rings. These protrusions invariably arrested at the stage of a tiny bud in agreement with the earlier reports (Ayscough et al., 1997). Strikingly, some latA-treated cells that failed to form a tiny bud exhibited an entirely unexpected behavior. They first underwent de novo Cdc42 polarization next to the previous division site followed by the recruitment of septins in the shape of an amorphous cap associated with the Cdc42 cluster (Figure 1C, region 1). As septins accumulated, the intensity of the Gic2PBD-RFP signal markedly decreased as described above. This, however, was not followed by the restructuring of the septins into the shape of a ring. Instead, after nearly disappearing, the Gic2PBD-RFP signal suddenly appeared outside of the septin cap, often adjacent to it (Figure 1C, region 2). The newly-formed Cdc42 focus began to accumulate septins, whereas the intensity of Cdc3-GFP signal at the original location diminished, indicating the relocation of septins to the new Cdc42 cluster. As substantial amount of the septins relocated, a second jump of the Gic2PBD-RFP signal to yet another position, unoccupied by septins, was observed in some cells (Figure 1C, region 3). We quantified this surprising “chasing” phenomenon by integrating the intensity of Gic2PBD-RFP and Cdc3-GFP signals within fixed regions of interest (Figures 1C and 1D). This quantification unambiguously demonstrated that the Cdc42 cluster indeed translocates on the cell membrane and is followed by the septin cap with a time delay of ∼12 min.

In both the control and the latA-treated cells, accumulation of septins at a particular membrane location was always preceded by a prominent focal activation of Cdc42. This supports the notion that Cdc42 acts upstream of, and is required for, septin recruitment (Iwase et al., 2006). Furthermore, the accumulation of septins was invariably accompanied by a decrease in the activity of the underlying Cdc42 cluster, suggesting that septins might suppress Cdc42 activity. This suppression effect is likely indirect. For example, it could be caused by septin-mediated recruitment of negative regulators or factors that inactivate the positive regulators of Cdc42. Regardless of the exact mechanism, repeated reestablishment of the Cdc42-GTP cluster away from the septin cap during chasing indicates that the suppression of Cdc42 activity is local and septin-dependent rather than controlled by a global cell-scale regulatory event, e.g., phosphorylation by CDK1.

### Modeling of Cdc42 Effector-Mediated Septin Recruitment and Septin-Mediated Negative Feedback

**Figure 2:**
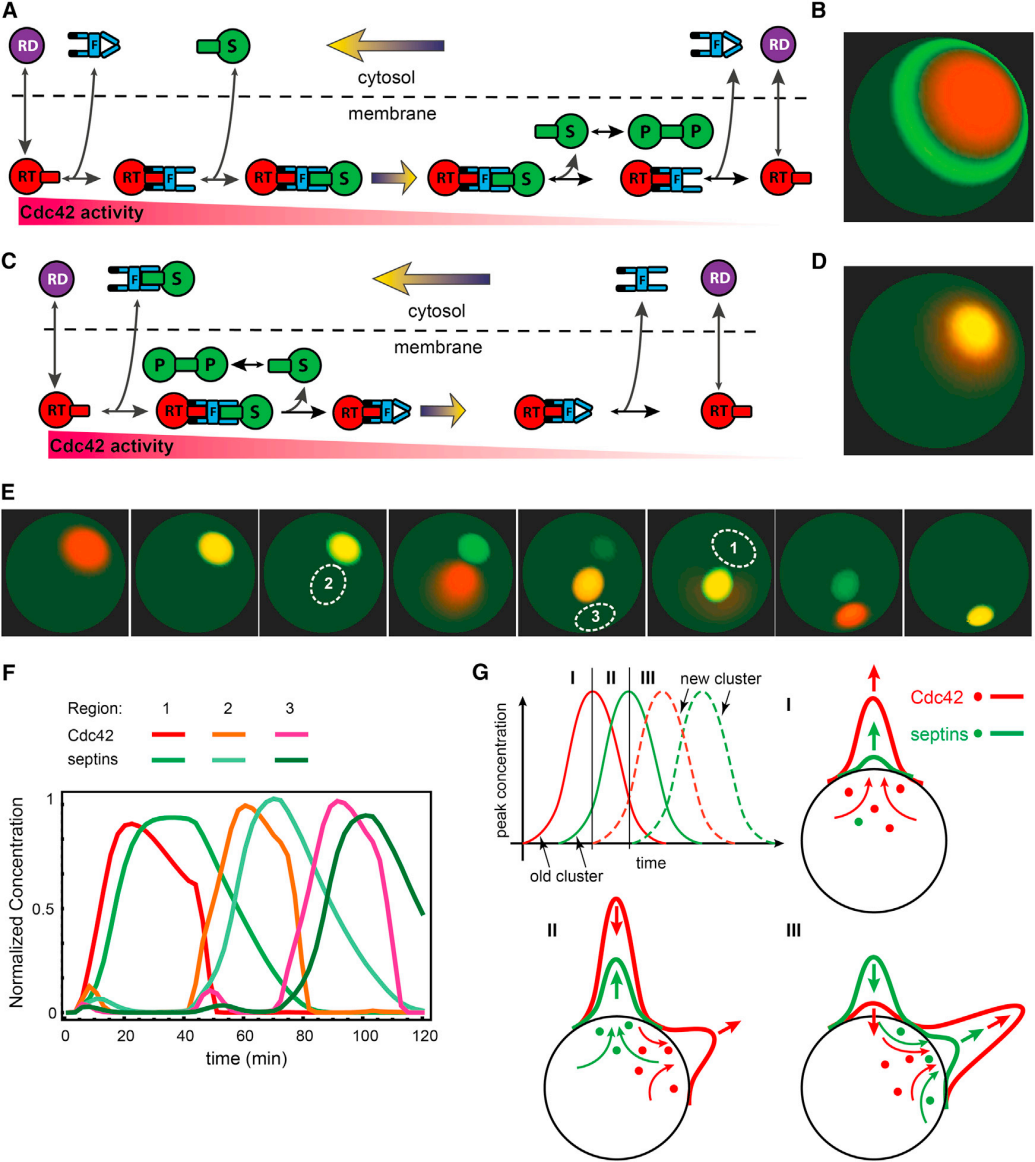
Model of Effector-Mediated Septin Recruitment Predicts Septin Cap Formation and Explains Chasing (A) Diagram of the model with exportin-like effector unlocked by Cdc42-GTP to bind septins. S, septin; P, polymeric septins; F, effector; RD, Cdc42-GDP; RT, Cdc42-GTP. Cdc42 activation, RD→RT, in the cluster center (left) and deactivation, RT→RD, on the cluster periphery (right) are shown schematically by arrows. Predominant direction of reactions is indicated by the size of arrowheads. Filled arrows represent diffusion gradients. (B) Broad septin ring (∼4 μm) forms in the model outlined in (A). (C) Diagram of the model with importin-like effector that releases septin upon binding to Cdc42-GTP. (D) Model sketched in (C) predicts a septin cap colocalized with the Cdc42 cluster. (E) Introduction of septin-mediated negative feedback into the model (C and D) recapitulates chasing phenomenon shown in Figure 1C. See also Movie S3. (F) Quantification of Cdc42-GTP and septins in the ROIs marked in (E). (G) Competition for the effector-septin complex explains chasing: (I) Cdc42-GTP recruits septins at the initial location; (II) waning old Cdc42 cluster still recruits septins more efficiently than the new cluster; (III) new cluster fully outcompetes the old.

Gic1 and Gic2, effectors of Cdc42, have been reported to participate in the recruitment of septins to the PBS (Iwase et al., 2006). Consistent with this, we observed that Gic1-GFP and Gic2-GFP arrived at the PBS after Cdc42-cluster formation at approximately the same time as septins (data not shown). To better understand the mechanism of Gic-mediated septin recruitment, we constructed a simple computational model that captured the most salient features of this process following the conceptual framework proposed earlier by us for the very emergence of the Cdc42 cluster (Goryachev and Pokhilko, 2008; Howell et al., 2009). The original model suggested a mechanism by which a cluster of activated GTPase on the membrane could recruit cytoplasmic proteins if they are transported as a cargo of a GTPase effector that undergoes multiple rounds of membrane-cytoplasmic shuttling. Effectors of small GTPases are frequently found in an autoinhibited conformation that is unlocked for the interaction with a target or cargo molecule only when the effector is bound to the activated form of the respective GTPase. For example, during nuclear export, exportins activated by Ran-GTP bind their cargo in the nucleus and release it in the cytoplasm (Cook et al., 2007). We hypothesized that Gics are recruited from the cytoplasm and activated by Cdc42-GTP in the PBS cluster, where they in turn recruit cytoplasmic septins (Figure 2A). Diffusively drifting down the concentration gradient on the membrane, Gic-septin complexes would eventually arrive at the periphery of the cluster where the Cdc42 activity is low allowing the complex to dissociate and release Gics back into the cytoplasm to perform additional rounds of recruitment while leaving the septins on the membrane enabling their polymerization. This hypothesis is attractive because it (1) explains the requirement for Cdc42 cycling between its nucleotide-bound states (Gladfelter et al., 2002), (2) allows a small quantity of an effector to translocate a large quantity of septins, and (3) suggests a self-sufficient mechanism by which the septin ring is templated by the Cdc42 cluster. Indeed, simulations of the model (Figures 2A and 2B; Supplemental Experimental Procedures) demonstrated formation of the septin ring in a broad range of parameter values. Despite this, we observed a number of striking contradictions between the model behavior and the experimental results. In the model, the septin ring encircled the entire Cdc42 cluster at all times and thus formed with a very large diameter of ∼4 μm (Figure 2B). On the contrary, in experiments, septins always accumulated first as an amorphous cloud in the center of the cluster, only eventually transforming into a ring whose internal diameter progressively increased to its final size of ∼1 μm. These inconsistencies indicated that our naive model was incorrect and prompted us to reconsider the proposed mechanism for Gic-mediated septin recruitment.

Organization of mammalian septins was shown to be regulated by the effectors of Cdc42, Borg1 and Borg3 (Joberty et al., 2001). Surprisingly, Cdc42-GTP inhibits Borg-septin interaction, suggesting that the Borgs bind septins in the absence of the GTPase and release septins when bound to Cdc42-GTP. This unusual mode of the GTPase-effector-cargo interaction has a well-characterized precedent. During nuclear import, importins bind their cargo in the cytoplasm and release it in the nucleus upon interaction with Ran-GTP (Cook et al., 2007). Recently, this interaction mode, in which an effector releases its cargo upon binding to the cognate GTPase, has been also reported for the Arl2 and Arl3 effectors PDEδ (Ismail et al., 2011) and UNC119 (Ismail et al., 2012; Wright et al., 2011). Thus, it is possible that Gics, like their mammalian functional counterparts, Borgs, may employ an importin-like mode of interaction with septins. Indeed, bacterially expressed Gic1 strongly interacted with septin complexes in the absence of Cdc42 (Figure S3). Unfortunately, for technical reasons, the effect of Cdc42-GTP on Gic1-septin interaction could not be determined with our methods. However, an independent study using electron microscopy demonstrated that high concentrations of Cdc42-GppNHp (nonhydrolyzable GTP analog) efficiently dissociated septin-Gic1 complexes in vitro (S. Raunser, personal communication). To explore this hypothesis further, we developed a distinct model in which septins and Gics can form complexes in the cytoplasm, in the absence of Cdc42-GTP. These complexes are then recruited to the center of the Cdc42 cluster where septins are released by the binding to Cdc42-GTP and the Gics are eventually recycled back to the cytoplasm (Figure 2C). This hypothesis possesses all the desirable properties of the exportin-like mechanism described above, except that it does not automatically generate the septin ring. Indeed, simulations demonstrated that in this model septins accumulate in the form of a circular cap (Figure 2D). Importantly, as a result of slowing of diffusion on the membrane due to the accumulation of septin polymers, the size of the underlying Cdc42 cluster was much smaller than that in the septin-free model (Goryachev and Pokhilko, 2008) or in the exportin-like model of septin recruitment (cf. Figure 2B). Thus, on its own, the importin-like mechanism of septin recruitment explains the size, but not the shape, of the nascent septin structure.

Next, we introduced a septin-mediated negative feedback to test whether this alone could explain the chasing phenomenon. Septins could inhibit Cdc42 activity passively, by crowding the membrane surface and excluding Cdc42 and its GEF Cdc24, and/or actively, by recruiting Cdc42 GTPase-activating proteins (GAPs). Passive exclusion mechanism alone was unable to recapitulate the experimental observations. However, addition to this passive mechanism of a GAP-dependent negative feedback resulted in a model with the behavior that remarkably resembled the chasing dynamics of septins (Figures 2E and 2F; Movie S3). The location of the new Cdc42 cluster depended on the molecular noise that was introduced into the model to account for the natural fluctuations in the molecular concentrations and reaction rates. Nevertheless, the chasing behavior itself was remarkably robust to the variation of the model parameters and addition of noise. Moreover, this model explained the remarkable discrete jumps of the septin cap observed in the experiment via the competition between the old and new Cdc42 clusters for the common cytoplasmic pool of the effector-septin complex, a property of the Cdc42 cluster proposed earlier as the cause of budding singularity (Goryachev and Pokhilko, 2008; Howell et al., 2009). During the short coexistence phase (II in Figure 2G), the old cluster continues to recruit septins more efficiently than the new one, thus generating the delay in the negative feedback that is necessary for the new cluster to establish itself. Together, these simulations suggest that a septin-mediated GAP-dependent negative feedback can explain the chasing phenomenon and, by extrapolation, the dynamics of Cdc42-GTP during the septin recruitment.

### Both Septins and Cdc42 GAPs Are Required for the Negative Feedback to the Cdc42 Activity

**Figure 3:**
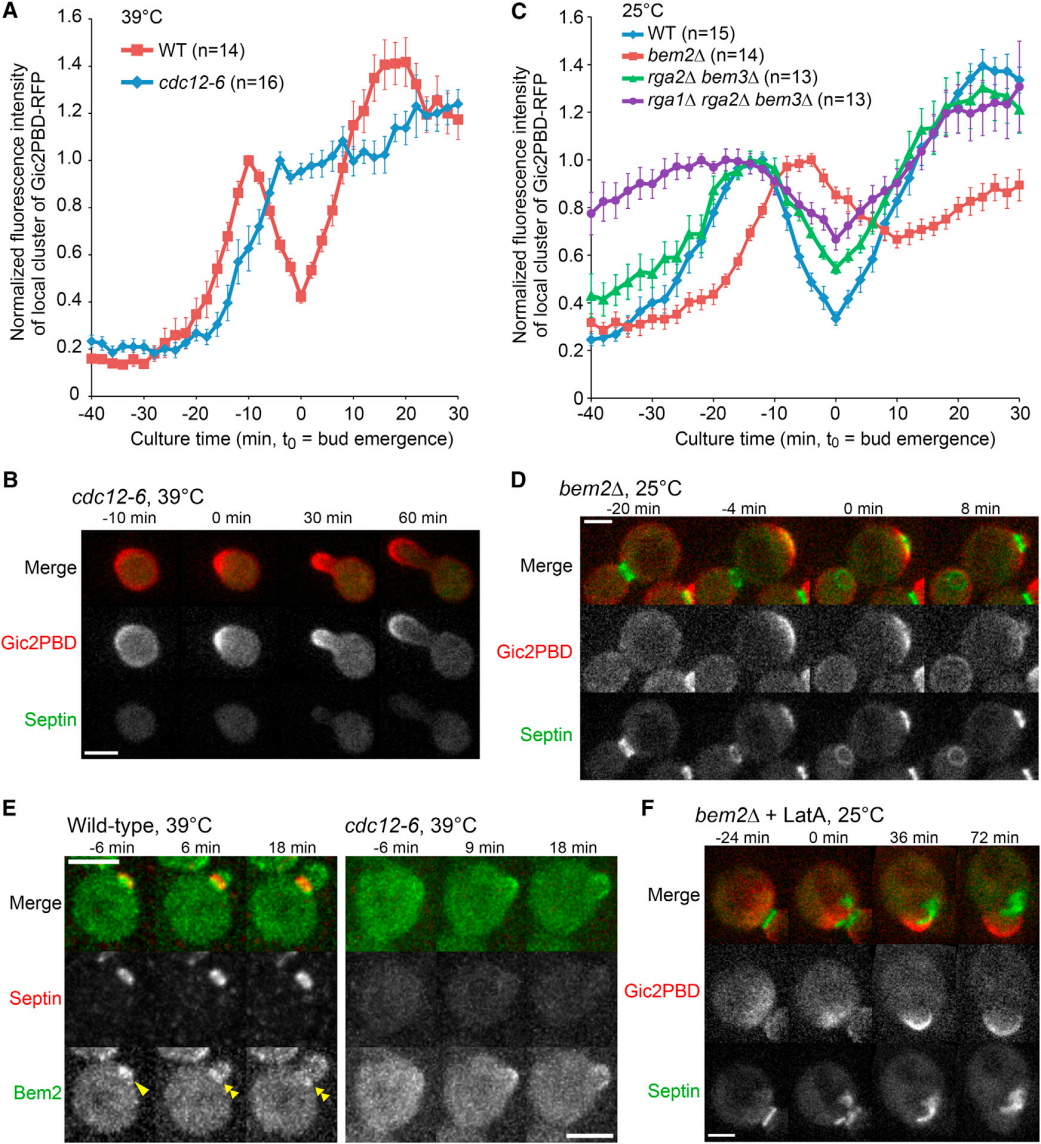
Septins Inhibit Cdc42 Activity in a GAP-Dependent Negative Feedback Loop (A) Negative-feedback regulation of Cdc42 requires septins. Gic2PBD intensity was quantified using a threshold method as in Figure 1B. Each point indicates average fluorescence intensity of Gic2PBD. Error bars represent SEM. (B) Hyphae-like polarized growth in a septin mutant *cdc12-6*. See also Figure S4. (C) Inhibition of Cdc42 activity depends on Cdc42 GAPs. Gic2PBD intensity was quantified using a threshold method as in Figure 1B. Each point indicates average fluorescence intensity of Gic2PBD. Error bars represent SEM. (D) Large Cdc42 clusters and wide septin rings formed in a Cdc42 GAP mutant (*bem2*Δ). See also Figure S4 and Movie S1. (E) Bem2 associates with septins at the PBS prior to bud emergence (single arrowhead) and with the septin ring afterward (double arrowheads). The localization of Bem2 at the bud neck, not the bud cortex, is fully dependent on septins (right panel). (F) Broad shmoo-like protrusions in latA-treated *bem2*Δ cells. See also Movie S2. Scale bars represent 3 μm.

To test whether the septin-mediated negative feedback does exist in the cell, we monitored Cdc42-GTP dynamics at the PBS in the temperature-sensitive septin mutant, *cdc12-6*. As expected, no septins were detected at the PBS at the nonpermissive temperature of 39°C. The mutant cells exhibited filamentous-like growth with large bright clusters of Gic2PBD-RFP signal prominently displayed at the tips of broad hyphae-like protrusions (Figures 3B, S4A, and S4B), in contrast to the well-defined buds formed at the permissive temperature (data not shown). Quantification of the Gic2PBD-RFP signal using the same algorithm as in Figure 1B demonstrated that the Cdc42 activity increased monotonously from its onset at the PBS to its ultimate level at the tip of an extending protrusion (Figure 3A). Addition of latA at 39°C substantially slowed, but did not abolish, polarized growth of broad protrusions (data not shown). These data suggest that septins are involved in a negative feedback loop to suppress the Cdc42 activity at the PBS during septin ring biogenesis.

To determine if Cdc42 GAPs are also involved in this feedback mechanism, we quantified the activity of Cdc42 in a panel of GAP deletion mutants and found that, in all mutants, the relative decrease in Cdc42 activity at the bud emergence was smaller than that in the wild-type (WT) cells (Figures 3C, 3D, and S4C–S4E), suggesting that these GAPs are involved in a negative feedback mechanism. As expected, deletion of several GAPs together produced a cumulative effect. Bem2 appeared to play a major role in the negative feedback because its activity alone accounted for ∼50% of the suppression effect (Figures 3C and 3D). Moreover, we found spatial association between septins and Bem2-GFP expressed from its native genomic locus both prior to and after bud emergence (Figures 3E, left, and S4F), which is consistent with an earlier report (Knaus et al., 2007; Schindelin et al., 2012). We also demonstrated, using *cdc12-6* mutant, that the localization of Bem2 to the bud neck, but not to the bud cortex, is completely dependent on the presence of septins (Figures 3E, right, and S4G). Among the cells undergoing polarization and bud emergence at 39°C, 44 out of 45 WT cells (∼98%) showed a clear neck localization of Bem2-GFP at the time of bud emergence, which remained there throughout the cell cycle. In contrast, none of the *cdc12-6* cells (0%, n = 37) showed any type of Bem2-GFP neck localization at the time of “bud emergence” or membrane protrusion. In addition, Bem2-GFP accumulation at the PBS was also decreased in nearly all the *cdc12-6* cells (Figures 3E, right, and S4G), suggesting that septins are involved in the recruitment of Bem2 to the PBS. As with the *cdc12-6* mutant, addition of latA produced no chasing in GAP deletion mutants and did not abolish polarized growth. In fact, some latA-treated cells exhibited broad vigorously growing shmoo-like protrusions with prominent Gic2PBD-RFP signal and septins that were either spread uniformly over the protrusion or found in disjoint fragments at the protrusion base (Figure 3F; Movie S2). Considering these results together, we conclude that both septins and Cdc42 GAPs are required for the negative feedback observed in the WT cells with or without latA.

### Polarized Exocytosis Could Hollow a Septin Cap into a Ring

**Figure 4:**
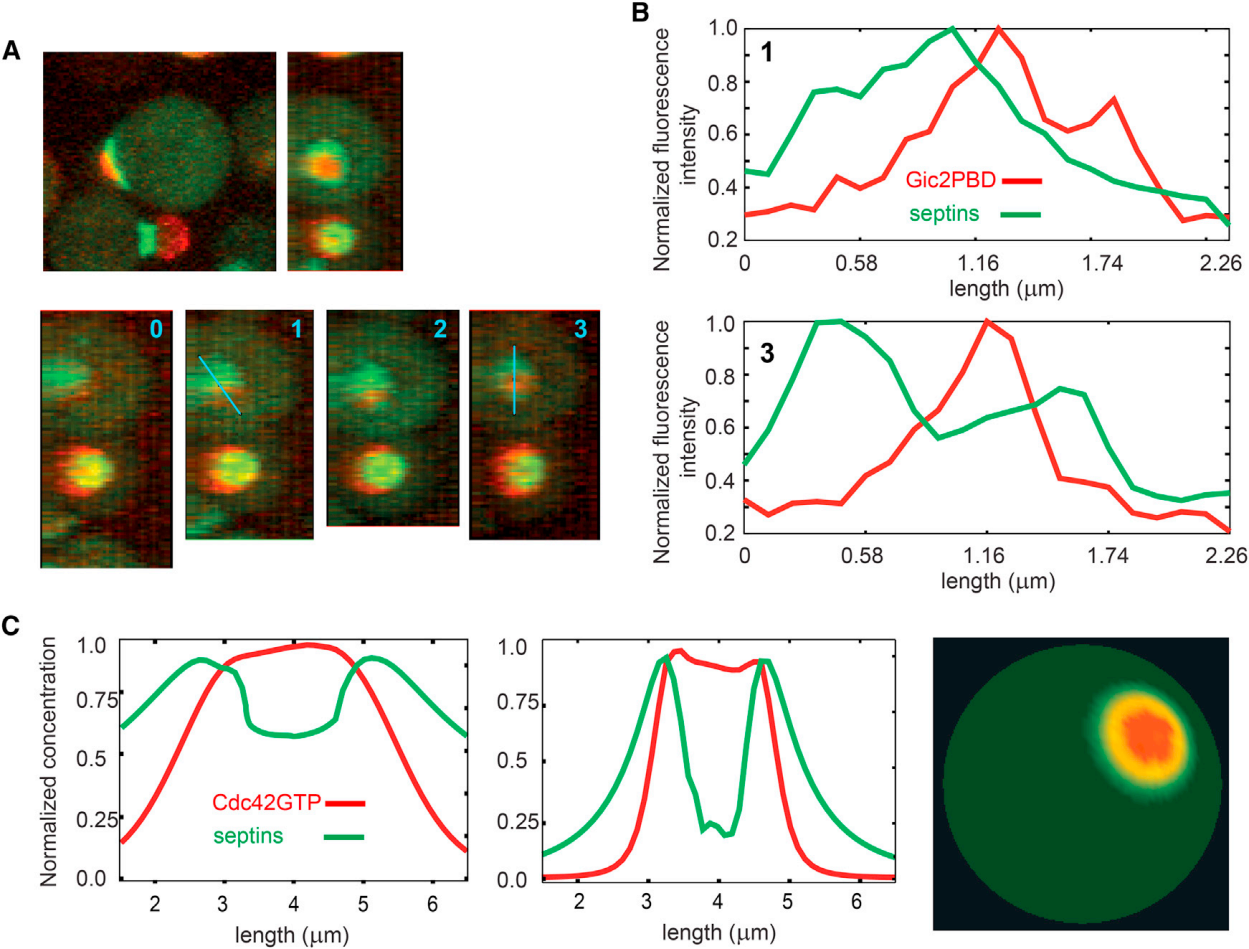
Polarized Exocytosis Could Transform a Septin Cap into a Ring (A) De novo emergence of a tiny bud in latA-treated wild-type cells. Top: side view and 3D reconstruction showing tiny bud (Gic2PBD-RFP) and the septin ring (Cdc3-GFP). Bottom: a series of four consecutive images (2 min apart) shows the septin cap-to-ring transition (3D reconstructions). (B) Spatial profiles of Gic2PBD and septin concentrations along the indicated in (A) line segments (blue), before and after the cap-to-ring transition. (C) Septin ring formation in the model with exocytosis. Shown are the concentration profiles at the initiation of septin cap hollowing (left), after the septin ring formation (middle), and the 3D view (right). See also Movie S4.

We carefully examined those latA-treated WT cells that formed a tiny bud with the characteristic “bull’s eye” morphology described above (Figure 4A) to precisely dissect the septin cap-to-ring transition. Heralded by a drastic change in the local membrane curvature, bud emergence events were distributed uniformly throughout the duration of imaging, suggesting that they were not dependent on latA degradation or expulsion from cells. As expected (Iwase et al., 2006), latA significantly slowed septin ring formation, which gave us an opportunity to observe it in detail. The first indication of the ring emergence took the form of a partial decrease of septin density in the center of the septin cap (Figures 4A and 4B) that progressively grew in width and amplitude until the cap was transformed into an irregular doughnut-shaped structure. Frequently, this initial depression in septin density was observed to temporarily reverse back, suggesting that, in addition to the ring-forming activity, there are also opposing forces that attempt to refill the ring opening with septins.

What could locally reduce the concentration of septins in the center of the cap? We noted that the maximum in the Cdc42-GTP profile was approximately colocalized first with the maximum of septin concentration and then with the depression in the septin concentration profile as it emerged (Figure 4B). Our results with latA-treated WT and mutant (*cdc12-6* and GAP deletion) cells are in agreement with those of the earlier studies, i.e., the disassembly of actin cables does not stop polarized exocytosis (Roumanie et al., 2005; Sahin et al., 2008; Yamamoto et al., 2010). In the absence of cables, polarized exocytosis could be driven by the interaction of Cdc42-GTP with its effectors, Sec3 and Exo70, both of which are subunits of the exocyst complex (He and Guo, 2009). Insertion of new membrane in the center of the Cdc42 cluster, where the probability of exocytosis would be the highest, could be responsible for both the septin ring opening and the protrusion of the bud. Indeed, insertion of vesicles would be expected to physically displace “old” membrane material outward. Molecules with appreciable diffusivity, such as Cdc42, septin complexes, and possibly even sparse septin filaments, would rapidly fill the “hole” created by the insertion of septin-free membrane, explaining the unstable, intermittent opening of the ring observed in some cells. In contrast, dense septin polymers with negligible diffusivity would remain attached to the underlying phospholipids of the plasma membrane and will be likely displaced together with them. Repeated insertion of vesicles targeted at the center of the Cdc42 cluster could simultaneously reduce the septin density in the center and increase it in the periphery, thus, generating a ring. Furthermore, several recent reports (Das et al., 2012; Freisinger et al., 2013) suggested that exocytic vesicles may deliver appreciable quantity of Cdc42 to the plasma membrane not only in *rdi1*Δ mutant, in which cytoplasmic transport of Cdc42 is prevented by deletion of the GDI protein, but also in WT cells. This transport could provide positive feedback to the activity of Cdc42 in the opening of the nascent ring.

To test the exocytic hypothesis of septin ring formation, we developed a modeling framework for simulation of exocytosis in a spherical cell (see Supplemental Experimental Procedures). In this more realistic and elaborate model, we assumed that vesicles are inserted randomly with a spatially-dependent probability that is increased by Cdc42-GTP and decreased by septins. Septins could suppress exocytosis independently of their inhibition of the Cdc42 activity either via nonspecific space-filling or by means of specific inhibitory interaction with SNAREs, as observed in platelets and neurons (Beites et al., 1999; Dent et al., 2002). Simulations show that introduction of exocytosis in the model transforms the septin cap into a ring (Figure 4C; Movie S4), highly similar to that observed in the experiments (cf. Figures 4A and 4C, right). Starting with a small depression caused by the insertion of just a few vesicles (Figure 4C, left), the septin structure progressed to a complete ring (Figure 4C, middle). Remarkably, this exocytosis-enabled model also reproduces the relocation of the Cdc42 activity completely within the ring enclosure where, insulated by the diffusion barrier of the ring, the Cdc42 cluster acquires concentration profile with sharp boundaries.

What is more important for the septin ring formation: physical insertion of membrane vesicles or their specific lipid content? In an attempt to address this question, we perturbed the plasma membrane lipid composition focusing on negatively charged phosphatidylserine (PS) and phosphatidylinositol 4,5-bisphosphate (PIP2) lipids. The flippase Lem3 has been shown to affect Cdc42-GDI interaction by influencing the asymmetric distribution of PS between the plasma membrane leaflets (Das et al., 2012). Our live-cell imaging, however, demonstrated that establishment of cell polarity as well as formation of the septin ring and tiny bud appeared normal in *lem3*Δ mutant (Figures S5A and S5B). To test the role of PIP2, a temperature-sensitive mutant *mss4-2* of the yeast plasma-membrane-specific phosphatidylinositol-4-phophate-5-kinase (PI4P5K) Mss4 was imaged for 2 hr at 37°C. Using GFP-2xPH(PLCδ1) probe, we detected an ∼2-fold decrease in the plasma membrane PIP2 after 30 min incubation at the restrictive temperature (Figures S5C and S5D). As expected, due to the loss of the membrane PIP2 (Bertin et al., 2012), the cytoplasmic pool of septins was increased in *mss4-2* cells compared to WT (Figures S5E–S5H). Nevertheless, *mss4-2* cells showed essentially normal budding that was often followed by depolarization of Cdc42 and cell lysis (data not shown). This robustness to perturbations of the membrane lipid composition suggests that it is the insertion of lipid vesicles per se that is primarily important for the formation of septin ring.

### Polarized Exocytosis Is Required for Septin Ring Formation

**Figure 5:**
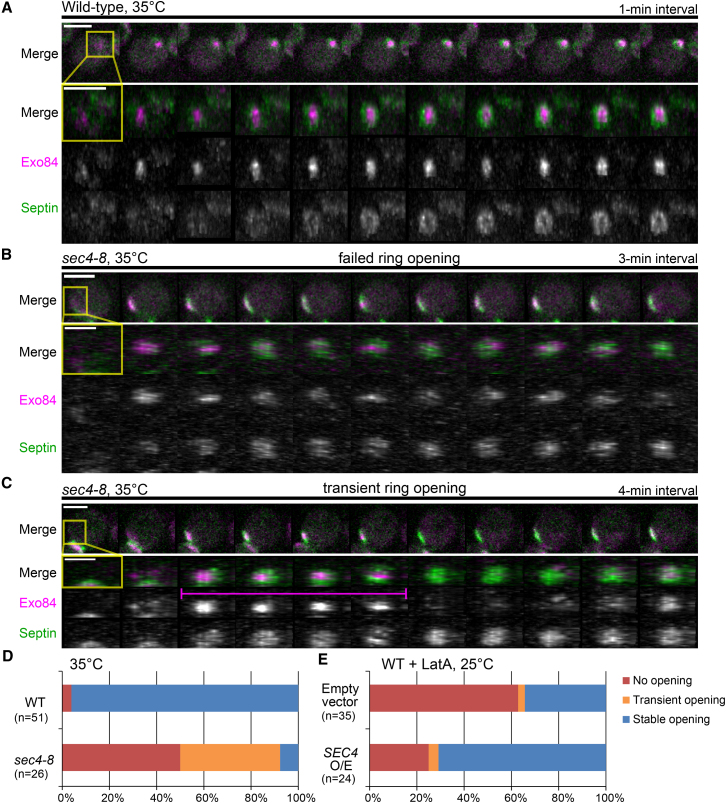
Robust Exocytosis Is Required for Stable Septin Ring Opening (A) Septin ring (Cdc3-mCherry) progressively forms around the zone of focused exocytosis (Exo84-GFP) in wild-type cells. Top panels: side-view image sequences of entire cells; bottom panels: en face-view image sequences generated by 3D-reconstruction of selected regions (yellow rectangles). See also Figure S6 and Movie S5. (B) An example of a failure to form a ring in *sec4-8* secretion mutant. Top panels: side-view image sequences of entire cells; bottom panels: en face-view image sequences generated by 3D-reconstruction of selected regions (yellow rectangles). See also Movie S5. (C) An example of a transient ring opening in *sec4-8* mutant. The duration of ring opening is marked by magenta bar. Top panels: side-view image sequences of entire cells; bottom panels: en face-view image sequences generated by 3D-reconstruction of selected regions (yellow rectangles). See also Movie S5. (D) Exocytic mutant *sec4-8* largely fails to produce stable septin ring opening. See also Figure S6 showing chasing in *sec4-8* cells. (E) Overexpression of *SEC4* rescues septin ring opening in latA-treated WT cells. See also Figure S6 and Movie S5. Scale bars represent 3 μm.

To test the exocytic hypothesis directly, we monitored the spatiotemporal dynamics of the exocytic machinery during septin ring formation and also determined the consequences of altering exocytic activity on septin ring opening. The exocytic activity was probed using GFP-labeled Exo84, an exocyst subunit that is known to exclusively decorate secretory vesicles (Boyd et al., 2004). As expected, Exo84-GFP was localized within the old septin ring during cytokinesis (data not shown). As this signal decreased, a new locus of exocytic activity was established at the PBS, which grew rapidly in intensity (Figure 5A; Movie S5). Simultaneously, the septins moved from the old ring to a new neighboring location, first appearing as a cloud and then as an imperfect ring surrounding the exocytic locus (Figure 5A; Movie S5). Again, rapid bud protrusion obliterated the fine details of this cloud-to-ring transition.

To decelerate secretion and, therefore, bud protrusion, we used a temperature-sensitive Rab GTPase mutant, *sec4-8*. At the nearly restrictive temperature of 35°C, bud formation was dramatically slowed or not observed at all. In ∼50% of the mutant cells, septins accumulated at the PBS but failed to form a ring (Figures 5B and 5D). Furthermore, some cells exhibited chasing (see Figures S6C and S6D) consistent with the conjecture that this phenomenon is caused by severe reduction in exocytic activity. In contrast, under the same conditions more than 90% of the WT cells formed a normal septin ring (Figures 5A and 5D). These data suggest that polarized exocytosis plays a critical role in septin ring opening. Strikingly, ∼40% of the mutant cells displayed pronounced fluctuation in exocytic activity at the PBS, which was accompanied by transient ring opening and closure (Figures 5C and 5D). In remarkable agreement with our predictions, a temporary decrease in the activity of exocytosis was frequently followed by the ring closure and reversion to a cap, demonstrating that continuous exocytosis is required to maintain the ring opening. Importantly, in both WT and mutant cells, the location of the nascent ring opening always correlated with the maximum of the exocyst signal. Reciprocally, the exocyst signal was progressively excluded from the areas with increasing septin density, thus corroborating our hypothesis that septins suppress exocytosis.

We reasoned that overexpression of Sec4, which is required for vesicle transport and tethering to the plasma membrane (He and Guo, 2009), might rescue the behavior of latA-treated cells by increasing the exocytic activity. Indeed, overexpression of Sec4 increased the probability of stable ring opening in latA-treated cells from 34% to 70% (Figures 5E, S6A, and S6B; Movie S5). Together, these results demonstrate that polarized exocytosis is required not only for the opening of the nascent septin ring but also for the maintenance of its opening during bud emergence.

### Septin Ring Diameter at Bud Emergence Correlates with the Size of Cdc42 Cluster Prior to Bud Emergence

**Figure 6:**
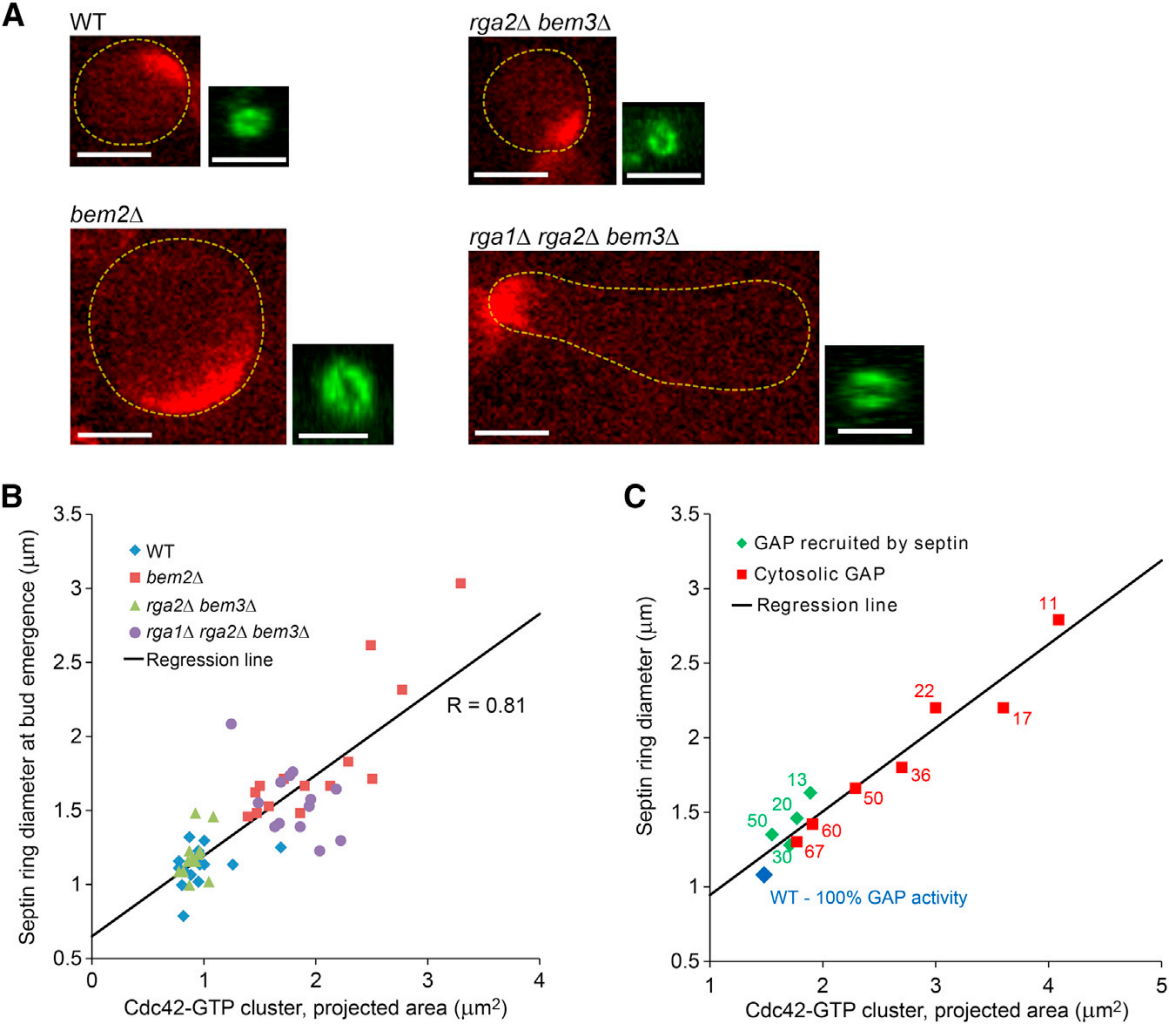
Diameter of Septin Ring Strongly Correlates with the Size of Cdc42-GTP Cluster (A) Representative images of Cdc42-GTP clusters at their peak activities before bud emergence (side-view, red) and newly formed septin rings at bud emergence (en face-view based on 3D-reconstruction, green). Cell outlines are indicated by the dotted yellow line. (B) Septin ring diameter correlates with the Cdc42-GTP cluster size at their peak activities in WT and Cdc42 GAP mutant cells. (C) Correlation between the septin ring diameter and Cdc42-GTP cluster size in model simulations with cytoplasmic and septin-bound GAP pools reduced to the fractions (%) indicated on the figure. Scale bars represent 3 μm.

Yeast and mammalian septins have been shown to spontaneously form circular structures with diameter 0.4–1.0 μm both in vitro and in vivo (Garcia et al., 2011; Kinoshita et al., 2002), suggesting that septin filament bundles may possess intrinsic curvature that determines the size of the septin ring. We noticed that the diameter of newly formed septin rings was significantly different in the WT and GAP mutant cells (Figure 6A). Comparative analysis of the ring diameter at bud emergence versus the cluster size of Cdc42-GTP during its peak activity at the PBS prior to bud emergence (Figure 6B) indicated that (1) despite variability between individual cells, there is a systematic difference between septin rings in distinct mutants, and (2) the ring diameter is highly correlated with the size of the Cdc42-GTP cluster at its peak activity, which coincides with the beginning of septin accumulation at the PBS. In the model, we separately reduced the dose of cytoplasmic and septin-bound GAPs to determine the role of each pool. This analysis (Figure 6C) shows that a decrease in the GAP activity results in an increase in the ring diameter as well as the size of the Cdc42-GTP cluster. Moreover, the cytoplasmic GAPs exert a more prominent effect on both as they, together with the GEF, define the size of the Cdc42-GTP cluster long before septin accumulation, whereas the septin-bound GAPs influence the diameter of the ring only after its formation. Based on our data (Figures 3C, 3E, 6A, and 6B), Bem2 is likely the major GAP in both the cytoplasmic and septin-bound pools, whereas Rga2 and Bem3 play minor and auxiliary roles.

In summary, these results suggest that the diameter of the septin ring is defined by the parameters of the biophysical processes responsible for its formation, in particular the size of the Cdc42-GTP cluster, rather than by some intrinsic properties of septin polymers, such as filament curvature.

## Discussion

In this study, by combining live-cell imaging and mechanistic whole-cell modeling, we propose a mechanism for Cdc42-controlled septin ring assembly in budding yeast. Unexpectedly, we find that septins provide negative feedback by inhibiting Cdc42-GTP via the recruitment of Cdc42 GAPs and possibly by affecting spatial distribution of other regulatory molecules. By decreasing exocytic activity, we reveal that Cdc42-mediated recruitment of septins results in the formation of a septin cap rather than a ring. We further demonstrate that the hollowing of the septin cap into a ring is generated by polarized exocytosis that is also controlled by Cdc42 activity. Furthermore, we show that this transition results in relocation of active Cdc42 and all of its downstream effectors into the septin ring enclosure. Thus, septin ring formation defines the emergence of distinct daughter cell identity and also prevents it from intermixing with that of mother.

### Feedback Loops between Cdc42 Activity, Septins, and Exocytosis Control Septin Ring Biogenesis

**Figure 7:**
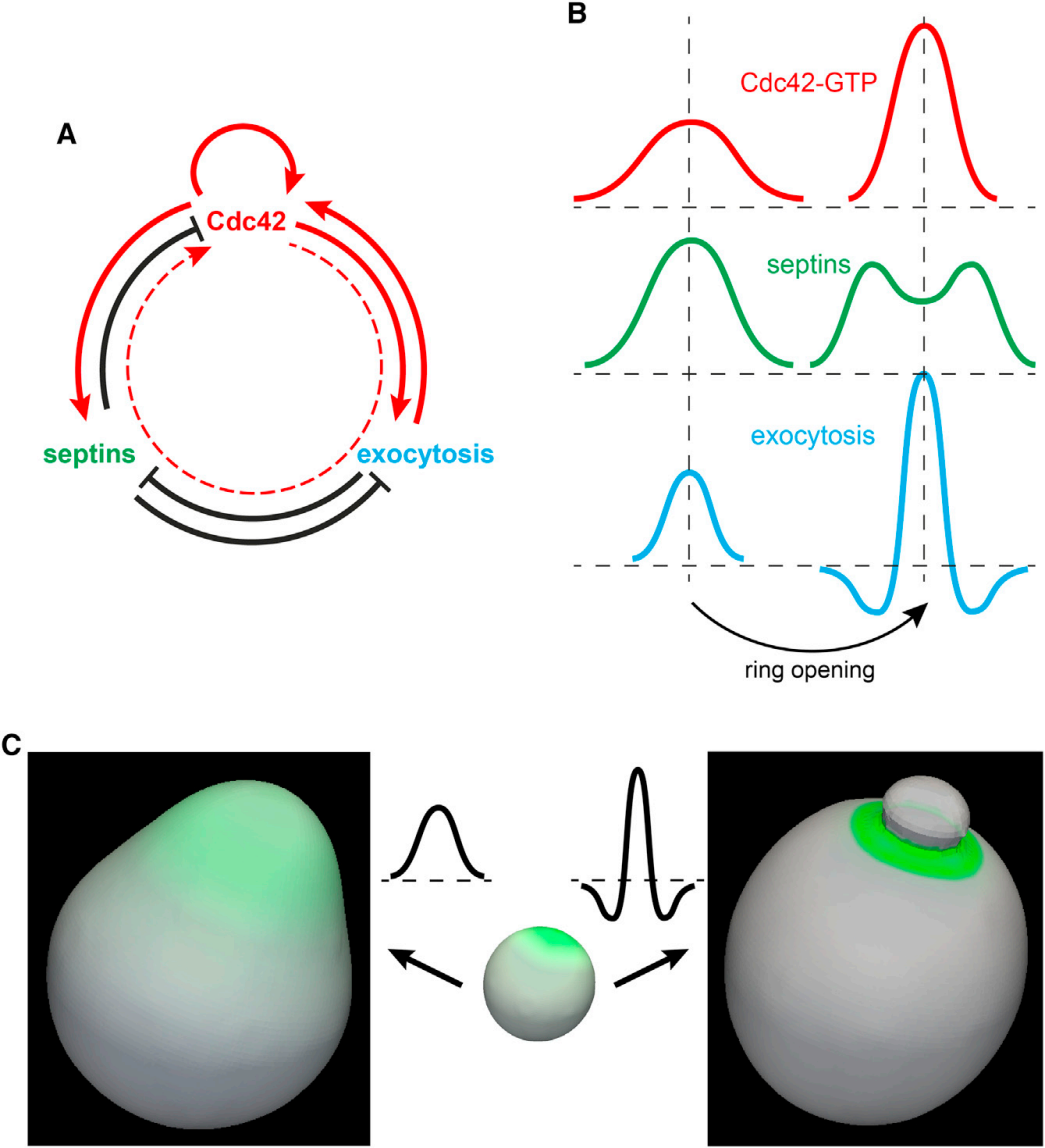
Complex Interplay between Cdc42-GTP, Septins, and Exocytosis Shape the Septin Ring and Yeast Bud (A) Positive and negative interactions between Cdc42-GTP, septins, and exocytosis described in text. (B) Schematic diagram of the septin cap-to-ring transition. (C) Distinct spatial profiles of exocytosis generate either a shmoo-like protrusion (left) or a small bud with a narrow neck and septin ring (right) from initially round cell with a septin cap. The level of isotropic exocytosis is shown by dashed lines.

We found that several interlinked feedback loops play important roles in septin ring assembly (Figure 7A). Unexpectedly, we found that septins, in a negative feedback loop, suppress the activity of the underlying Cdc42 cluster. This feedback mechanism completely depends on septins (Figures 3A and 3B) and, at least partially, on Cdc42 GAPs (Figures 3C and 3D) that associate with septins at the PBS (Figure 3E).

Cdc42-GTP controls polarized exocytosis indirectly through formin-generated actin cables (Chen et al., 2012) and directly through interactions with the exocyst (He and Guo, 2009). At the same time, exocytosis boosts Cdc42 activity by delivering Cdc42 to the plasma membrane. Our study revealed a mutually antagonistic interaction between septin accumulation and exocytosis. Following the hypothesis that insertion of the exocytic membrane may effectively dilute septin accumulation, we directly demonstrated a negative spatial correlation between the local septin density and the intensity of exocytosis (Figure 5C). Reciprocal inhibition of exocytosis by the accumulation of septins, as suggested earlier in mammalian cells, is also supported by our imaging results. Furthermore, our model simulations show that this negative interaction is, in fact, essential for the formation of a well-defined septin ring. Abrogation of this antagonistic interaction in the model invariably resulted in progressive fragmentation of the nascent ring due to random exocytic events that occur within the ring.

Negative feedback plays a key role in the regulatory networks of biological systems and has been implicated in generating rapid in-place oscillations of the Cdc42 cluster (Howell et al., 2012) and traveling waves of the Cdc42 activity (Ozbudak et al., 2005), both observed in a yeast mutant (*rsr1*Δ) lacking spatial landmark-directed polarization. Unlike the latter phenomenon that was significantly reduced by actin depolymerization, translocation of the Cdc42 cluster in our study was observed only when the function of the exocytic machinery was compromised.

### Highly Focused Exocytosis Is Crucial for Septin Ring Formation

Our study also uncovered an unappreciated role of polarized exocytosis in the spatial control of septin-mediated inhibition of Cdc42 activity at the PBS. In WT cells, frequent and spatially focused exocytosis could continuously displace the accumulating septins centrifugally, thus making the ring the only well-resolved septin structure observed at the PBS (Chen et al., 2011). In contrast, reduced frequency of targeted exocytosis in latA-treated cells allows septins to accumulate at the PBS in the shape of a cap and inhibit Cdc42 activity within it, thus causing dissipation of the underlying Cdc42 cluster. Based on the rate of growth of total cell surface in our experiments, latA reduces the spatially-averaged rate of exocytosis by at least 5-fold. This is likely a gross underestimation because, in cells untreated with latA, cell surface growth is opposed by endocytosis whose rate could not be determined and thus accounted for in our simple estimate.

The disassembly of the cluster at the original PBS and its reestablishment at a new location followed by septin relocation to this site constitute the chasing phenomenon as seen in some latA-treated cells. Observation of chasing in the *sec4-8* mutant at the nonpermissive temperature (Figures S6C and S6D) further suggests that impaired exocytosis, not other effects of latA, is the cause of chasing. In the model, chasing is observed not only in the total absence of exocytosis but also with a weak exocytosis. The increase in the frequency of exocytosis first slows and then completely stops chasing indicating that, for the stable formation of a septin ring, the intensity of exocytosis must exceed certain threshold.

Does chasing have any adaptive function? We speculate that chasing may improve survival rate of cells with decreased exocytic activity. Reduced rate of exocytosis could potentially cause bud malformation resulting in failed cytokinesis and cell death. Septin-mediated negative feedback ensures that cells with subthreshold exocytic activity, rather than allowed to proceed into later developmental stages with a potentially defective bud, are redirected into the abortive pathway leading to the complete disassembly of the unsuccessful PBS. The following reestablishment of the PBS at a new location offers the cell a new chance to form a bud. Indeed, with the overall reduction in the average rate, exocytosis becomes more stochastic and thus uneven in time (Figure 5C). Therefore, it is plausible that concomitant with a subsequent attempt at the PBS establishment, a sudden burst of exocytosis may provide just enough membrane to generate a stable ring opening. In support of this hypothesis, in both latA-treated WT and *sec4-8* mutant cells, we observed septin ring formation that followed after a variable period of chasing. Together, our experimental data and modeling suggest that highly focused exocytosis is critically important for septin ring formation.

### Cdc42 and Septin Ring Define Distinct Cell Identity of the Nascent Bud

Using the interactions depicted in Figure 7A, the exocytosis-dependent transition from a septin cap to a ring can be explained using the diagram in Figure 7B. Insertion of new membrane at the peak of exocytosis probability function (blue) causes septin density to change from a unimodal profile to a profile with a depression in the middle. This change partially relieves the septin-mediated repression of Cdc42 activity in the center but increases it on the sides of the cluster causing the profile of the Cdc42 cluster to become even narrower. Delivery of Cdc42 with the vesicles can also contribute to the increase of Cdc42 activity within the nascent ring opening. Because the probability of exocytosis is positively regulated by Cdc42-GTP and, independently, negatively by septins, its spatial profile changes as shown in Figure 7B. Thus, the probability of exocytosis in the center of the septin depression increases further whereas in the area occupied by the nascent septin ring it drops. Subsequent exocytic events further amplify Cdc42 activity inside the forming ring, leading to its dramatic rise that is robustly observed in our experiments (Figures 1A, 1B, and 4A) and fully reproduced in our model behavior (Figure 4C and 4D). This increase in the activity of Cdc42 on the membrane of the nascent tiny bud can be explained by the synergy of the direct positive feedback due to the exocytosis-dependent Cdc42 transport and the indirect exocytosis- and septin-dependent positive feedback loop characterized in this study (Figure 7A, dashed line).

The exclusive localization of activated Cdc42 within the nascent septin ring results in inevitable relocation into the bud of all Cdc42 effectors and their interactors. Because the exocytic activity is also confined to the bud, the enclosure of the septin ring defines a membrane domain with distinct lipid and protein composition. This membrane domain is a first manifestation of the unique daughter identity that is prevented from remixing with that of mother by the septin diffusion barrier.

### Septin Ring and Bud Neck Are Shaped by a Common Morphogenetic Process

The interplay between the morphology of the septin ring and the cell shape has long attracted attention in the field (Caviston et al., 2003; Gladfelter et al., 2005). We observed that cells deleted for Cdc42 GAPs formed broad dome-shaped, shmoo-like protrusions in the presence of latA (Figure 3F; Movie S2). Septins were either distributed uniformly over the entire protrusion or concentrated into irregular fragments at the base of the protrusion. Such phenotypes can be explained using the relationships established in our study (Figures 7A and 7B). The GAP mutant (*bem2*Δ or *rga1*Δ *rga2*Δ *bem3*Δ) cells formed large Cdc42 clusters with abnormally high total activity of the GTPase (Figure 6). Consequently, septins recruited to these clusters formed broader and therefore less dense caps, providing weaker suppression of the Cdc42 activity than in WT cells. In the absence of actin cables that focus the spatial distribution of exocytosis, insertion of new membrane is directed largely by the broad spatial profile of Cdc42-GTP. Membrane vesicles randomly inserted within the Cdc42 cluster further widen the cluster and dilute the septin density. Moreover, the generation of two distinct zones, the septin-dense “ring” that excludes exocytosis and the exocytosis-generated “hole” that excludes septins, does not occur. Instead, the broad initial distribution of Cdc42-GTP and exocytosis generate a protrusion in which the Cdc42 activity, septin density and exocytosis remain spatially distributed as shown in the left column of Figure 7B.

To test our hypothesis that the shape of the spatial profile of exocytosis is responsible for the morphological dichotomy between a “bud” that is separated from its mother by a slim hourglass-shaped neck and a tapered conical “shmoo,” we resorted to a computational model that permits evolution of the cell shape with time (see Supplemental Experimental Procedures for details). In these simulations, starting with a spherical cell with a polar septin cap, the cell membrane was continuously expanded using two distinct spatial profiles shown in Figure 7C. A characteristic “neck” with a diameter narrower than that of the expanding “bud” was formed only under the assumption of a ring-shaped zone where the probability of exocytosis was practically negligible. In these simulations, septins were segregated into this zone, forming a septin-dense ring whereas the membrane of the nascent bud was essentially septin-free. In contrast, the bell-shaped profile of membrane expansion led to the generation of a shmoo-like protrusion with continuously distributed septin density. Thus we conclude that the lack of a well-defined septin ring results in the loss of both the distinct membrane identity and the bud-like shape of a nascent protrusion.

In this study, we provide a coherent systemic model describing how an initially homogeneous cell can give rise to two distinct membrane identities that are then amplified and transformed into two different cellular fates. In its present form, the model captures only the most salient features of this process and, therefore, is inevitably incomplete. Many additional factors not considered here explicitly, such as membrane phospholipids, other small Rho GTPases, cell wall and factors that affect its remodeling, are likely to contribute to the emergence of bud identity and elucidation of their role is the subject of future work. The main driver of eukaryotic cell polarity, Cdc42, plays dual role in this process by both defining the identity of daughter membrane and creating the diffusion barrier in the form of a septin ring. Both functions are vitally important for the robust segregation of mother and daughter cellular identities. Future studies will likely reveal further contributions of polarity regulators and, in particular, Cdc42, in the differential distribution of cell-fate factors in other asymmetrically dividing cells.

## Experimental Procedures

### Live-Cell Imaging

Cells were cultured in synthetic complete (SC)-dropout media (a specific amino acid or uracil was omitted) to exponential phase at 25°C and then embedded in a layer of medium solidified with 1.2% low-melting-temperature agarose (FMC BioProducts) in a polylysine-coated glass bottom dish (MatTek). In some cases, cells were pretreated with 100 μM latrunculin A (latA) or high temperature (ranging in 35°C–39°C) before time-lapse analysis. During live-cell imaging, the temperature of the sample was kept constant in an environmental chamber (DH-35, Warner Instruments). Image acquisitions were performed on a microscope (Olympus, IX71) with a spinning-disk confocal scan head (Yokogawa, CSU10) and a 100× objective lens (Olympus, 1.4NA, Plan S-Apo oil immersion). An EMCCD camera (Hamamatsu Photonics, ImagEM, C9100-13) was used for image acquisition. Image acquisition was controlled by MetaMorph version 7.7 (Molecular Devices). Two diode lasers (488 nm for GFP and 561 nm for RFP) set in a laser integrator (Spectral Applied Research) were used for excitation illumination. Acquisition intervals were set in a range from 1 to 3 min.

### Image Processing

Image processing and analysis were performed using Fiji (Schindelin et al., 2012). For quantification of fluorescence intensities, image sequences generated by average projection were used. To quantify the intensity of a local cluster of Gic2PBD, a custom-made Fiji macro based on threshold method (see Supplemental Experimental Procedures) was used (script available on request). To quantify the septin-ring-opening phenotypes, we analyzed 60 min movies (experiments performed at 35°C) or 80 min movies (experiments performed at 25°C). We counted only the cells that showed Exo84-GFP spot newly formed at the PBS within the duration of the movie.

### Computational Modeling

Modeling has been performed using coupled reaction-diffusion equations representing all biochemical reactions, membrane-cytoplasmic shuttling and diffusion of molecules on the membrane. Due to the dramatic difference between the diffusion coefficients of species in the cytoplasm and on the membrane, diffusion in the cytoplasm was considered instantaneous. The biochemical core of the model (Goryachev and Pokhilko, 2006) describes nucleotide cycling of Cdc42 under the control of its GEF, Cdc24, and several GAPs. We considered two cytoplasmic pools of GAPs: that can reversibly bind septin polymers and that do not interact with septins. According to our data, some GAPs, e.g., Bem2, are likely to play prominent role in both pools, therefore molecular identities of GAPs were not resolved separately. Membrane-cytoplasmic shuttling of Cdc42 and Cdc24 was treated as in the earlier published model (Goryachev and Pokhilko, 2008). Septins were recruited to the membrane by Cdc42-GTP via an effector that interacted with septin monomers as a cargo. Two alternative models of GTPase-effector-cargo interaction were explicitly considered and compared to the experiment. In the exportin-like mechanism, the effector first binds Cdc42-GTP and is then activated to recruit cytoplasmic septins, whereas in the importin-like mechanism, cytoplasmic septin-effector complexes are recruited by Cdc42-GTP whose binding to the effector releases septins from the complex. Free septin monomers on the membrane could reversibly polymerize or recycle back to the cytoplasm. Details of all model equations are given in the Supplemental Experimental Procedures and the reaction constants and species concentrations are presented in the Supplemental Experimental Procedures, section 7.1.

Model reaction-diffusion equations were solved numerically using a standard finite volume method on a spherical surface representing the inner leaflet of the budding yeast plasma membrane. Although the majority of model reactions can be considered in the deterministic approximation, molecular noise plays an important role in the spontaneous initiation of Cdc42-GTP cluster formation (Goryachev and Pokhilko, 2008), which, in the present study is relevant, for example, as the inherent component of the chasing phenomenon. Therefore, the reaction of spontaneous activation of Cdc42 was simulated using a standard Langevin approach.

Exocytosis of vesicles was considered a Poisson process with a spatially-dependent rate positively regulated by Cdc42-GTP and negatively by the septin polymer density. Total intensity of exocytosis was estimated from the experimental data. Insertion of exocytic vesicles onto the spherical surface was performed by a numerical algorithm that faithfully represents expansion of the surface and conserves mass of all species on the membrane and vesicle.

To simulate our model on the evolving surface of the protruding cell, we utilized a distinct computational approach based on the Lagrangian particle method (Bergdorf et al., 2010). In this flexible approach, molecular concentrations are discretized on a set of disjoint computational particles rather than a static mesh and the evolving membrane surface is represented by a set level function that moves in 3D space with the velocity defined by the spatially-dependent probability of exocytosis. Detailed description of all numerical methods and their computational implementation is presented in the Supplemental Experimental Procedures.

## Supplemental Information

Document S1. Supplemental Experimental Procedures and Figures S1–S6

Movie S1. Dynamics of Cdc42-GTP and Septins during Cell Polarization in Wild-Type and Cdc42 GAP Mutant Cells, Related to Figures 1A, 3D, and S4 A wild-type (WT) strain YEF6699-1 and Cdc42 GAP mutant strains YEF6667-1 (*bem2*Δ), YEF6700-1 (*rga2*Δ *bem3*Δ) and YEF6692-1 (*rga1*Δ *rga2*Δ *bem3*Δ) were filmed at 25°C. Scale bar, 3 μm.

Movie S2. Dynamics of Cdc42-GTP and Septins during Cell Polarization in LatA-Treated Wild-Type and Cdc42 GAP Mutant Cells, Related to Figures 1C and 3F A WT strain YZT292-1 and a Cdc42 GAP mutant strain YZT393 (*bem2*Δ) were filmed at 25°C in the presence of 100 μM latA. Scale bar, 3 μm.

Movie S3. Simulation of Chasing Phenomenon in the Model, Related to Figure 2 Local concentrations of septins and Cdc42-GTP are color-coded as shades of green and red, respectively.

Movie S4. Formation of a Septin Ring in a Model with Polarized Exocytosis, Related to Figure 4 Local concentrations of septins and Cdc42-GTP are color-coded as shades of green and red, respectively.

Movie S5. Dynamics of Exocyst and Septins during Cell Polarization in Wild-Type and Exocytic Mutant Cells and in LatA-Treated Wild-Type Cells Harboring an Empty Vector or a SEC4-Overexpression Plasmid, Related to Figure 5 (0:00) A WT YEF5862-1 and an exocytic mutant YEF6497-1 (*sec4-8*) strain were filmed at 35°C. Scale bar, 3 μm. (0:21) A WT strain harboring an empty vector (YEF6640-1) and a wild-type strain harboring a *SEC4*-overexpression plasmid (YEF6641-1) were filmed at 25°C in the presence of 100 μM latA. Scale bar, 3 μm.
