# Supplementary material for: Daughter Cell Identity Emerges from the Interplay of Cdc42, Septins, and Exocytosis
Source: Dev Cell. 2013 Jul 29;26(2):148–61. doi: 10.1016/j.devcel.2013.06.015 (PMC3730058; doi:10.1016/j.devcel.2013.06.015)
Supplement: Document S1. Supplemental Experimental Procedures and Figures S1–S6 [file mmc1.pdf]

Developmental Cell, Volume *26*

Supplemental Information

Daughter Cell Identity Emerges from the Interplay of Cdc42, Septins, and Exocytosis

Satoshi Okada, Marcin Leda, Julia Hanna, Natasha S. Savage, Erfei Bi, and Andrew B. Goryachev

Inventory of Supplementary Materials

Supplementary Figures and Figure Legends

Figure S1. Development of improved Cdc42-GTP probe, Related to Figure 1.

Figure S2. Definition of the Cdc42-GTP Quantification Method, Related to Figure 1.

Figure S3. Gic1 Binds to Septin Complexes in the Absence of Cdc42-GTP, Related to Figure 2.

Figure S4. Suppression of Cdc42 Activity by Newly Recruited Septins and Cdc42 GAPs, Related to Figure 3.

Figure S5. Perturbations of the Plasma Membrane Lipid Composition Do Not Cause Obvious Defects in Septin Ring Assembly and Bud Emergence, Related to Figure 4.

Figure S6. Effect of *SEC4* Overexpression on Septin Ring Assembly in LatA-treated Cells and Chasing Phenomenon in an Exocytic Mutant, Related to Figure 5.

Supplementary Movie Legends

Movie S1. Dynamics of Cdc42-GTP and Septins during Cell Polarization in Wild-type and Cdc42 GAP Mutant Cells, Related to Figures 1A, 3D, and S4.

Movie S2. Dynamics of Cdc42-GTP and Septins during Cell Polarization in LatA-treated Wild-type and Cdc42 GAP Mutant Cells, Related to Figures 1C and 3F.

Movie S3. Simulation of Chasing Phenomenon in the Model, Related to Figure 2.

Movie S4. Formation of a Septin Ring in a Model with Polarized Exocytosis, Related to Figure 4.

Movie S5. Dynamics of Exocyst and Septins during Cell Polarization in Wild-type and Exocytic Mutant Cells and in LatA-treated Wild-Type Cells Harboring an Empty Vector or a *SEC4*-overexpression Plasmid, Related to Figure 5.

Supplementary Experimental Procedures

Supplementary References

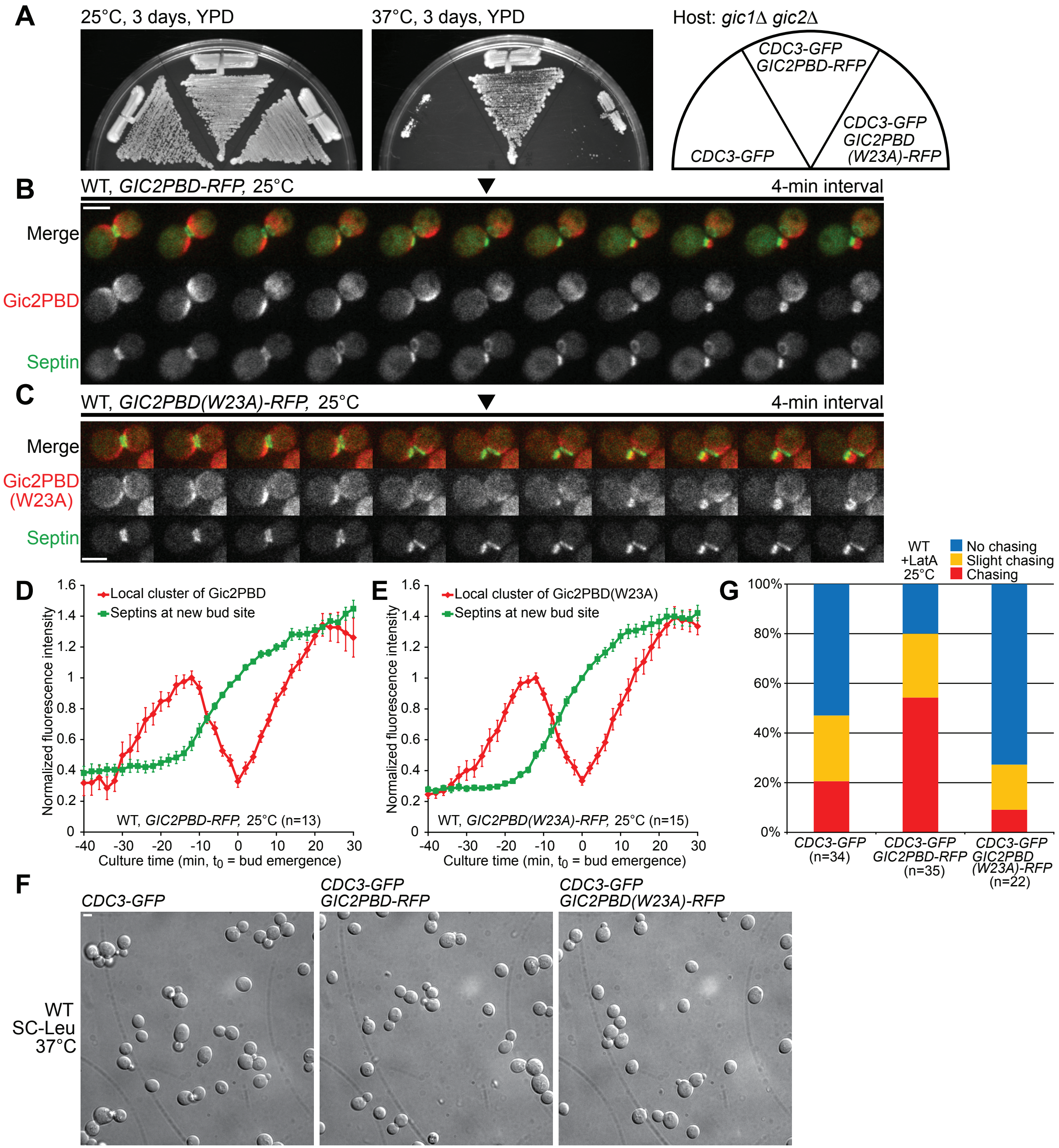

Figure S1. Okada *et al.*

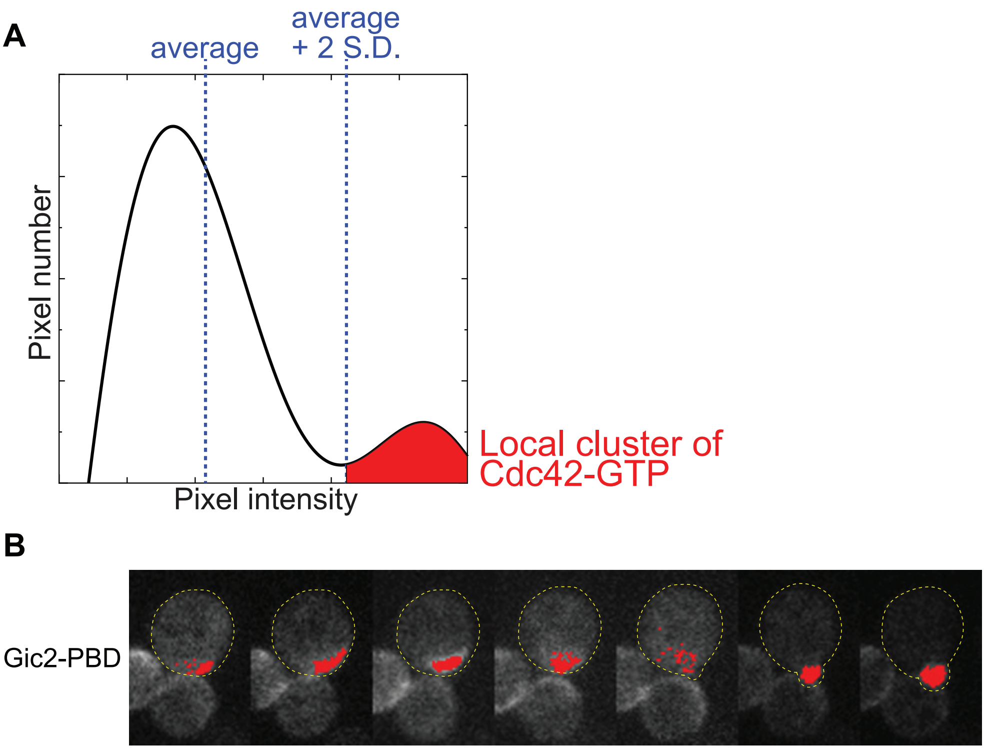

Figure S2. Okada *et al.*

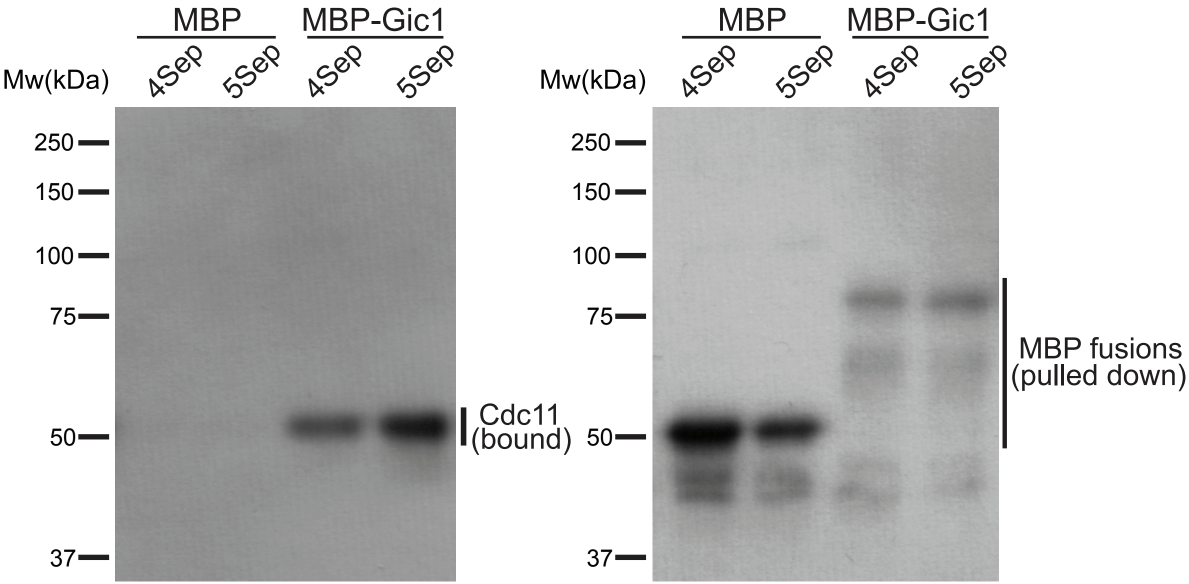

Figure S3. Okada *et al.*

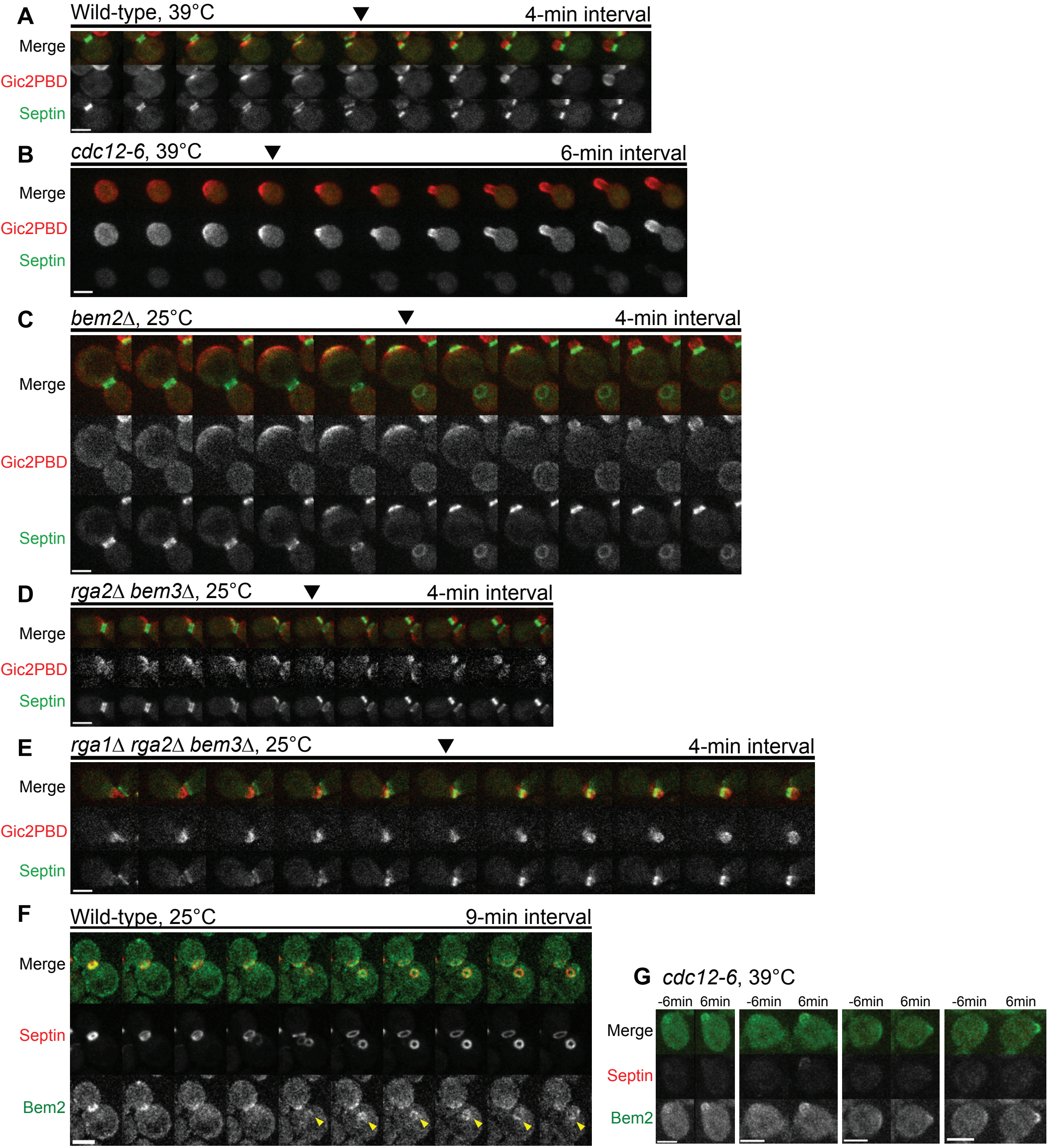

Figure S4. Okada *et al.*

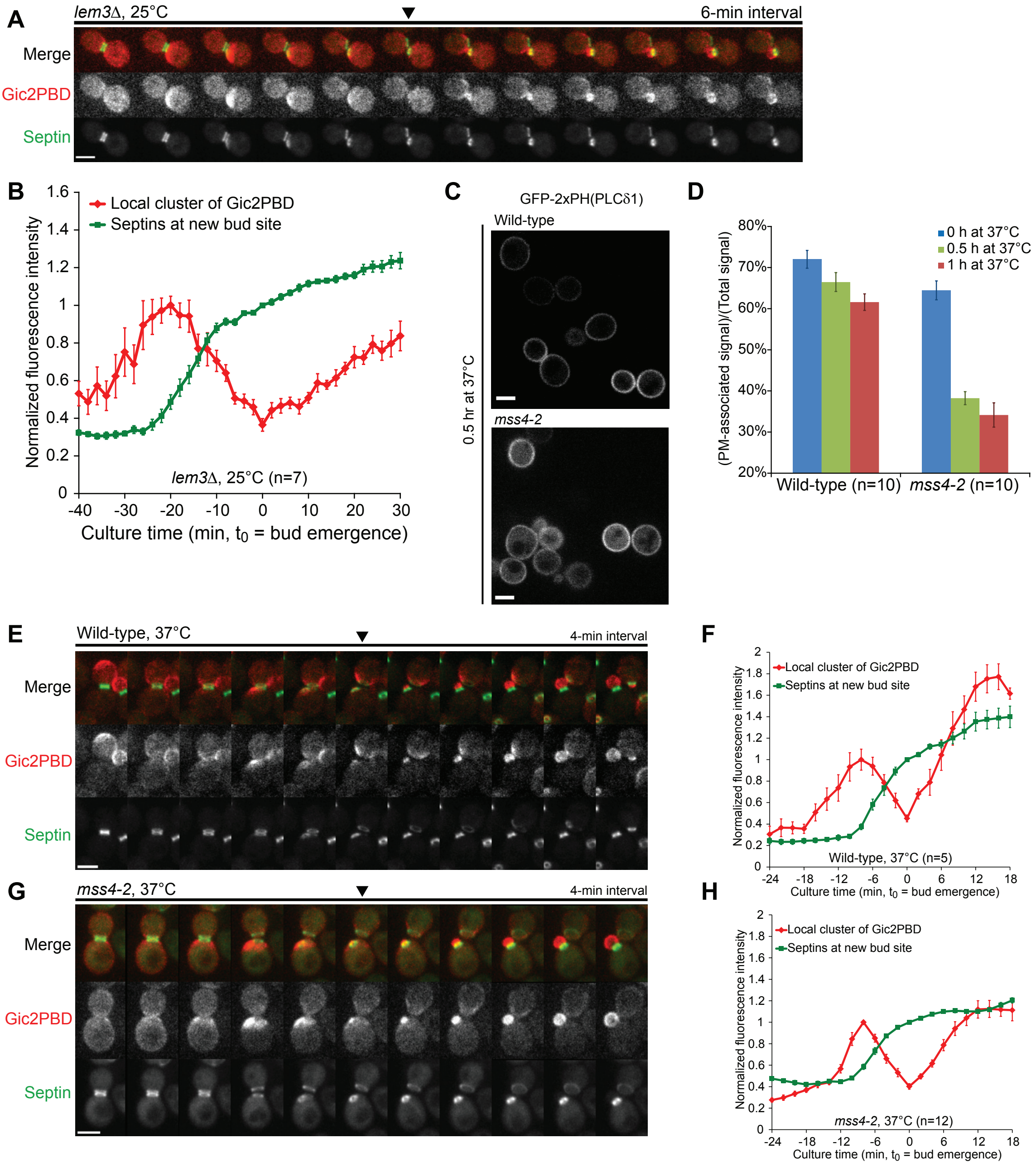

Figure S5. Okada *et al.*

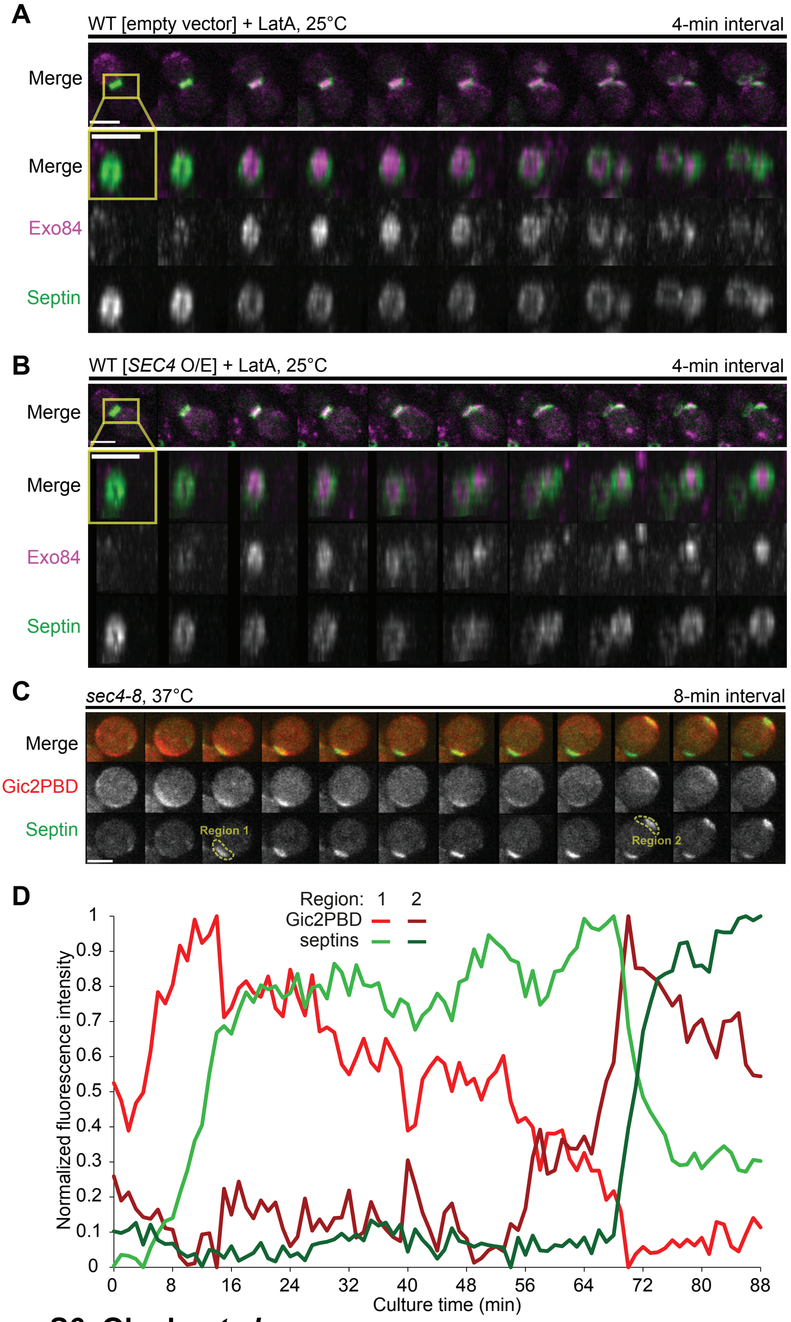

Figure S6. Okada *et al.*

Supplementary Figure Legends

Figure S1. Development of improved Cdc42-GTP probe, Related to Figure 1

(A) The improved Cdc42-GTP probe *GIC2PBD(W23A)-RFP* does not complement *gic1*Δ *gic2*Δ cells. The strains YEF5927-2 (*gic1*Δ *gic2*Δ *CDC3-GFP*), YEF5981-1 (*gic1*Δ *gic2*Δ *CDC3-GFP*) carrying the old probe, *GIC2PBD-RFP*, and YEF6670-1 (*gic1*Δ *gic2*Δ *CDC3-GFP*) carrying the improved probe were grown on YPD plates at 25°C (left) or 37°C (center) for 3 days. The right panel indicates the relevant genotypes.

(B-C) The two probes display similar dynamics during the cell cycle. Figures 1A and 1B are reproduced here as panels C and E, respectively, to facilitate the comparison of two probes. Cells of haploid wild-type (WT) strains YZT292-1 (*CDC3-GFP GIC2PBD-RFP*) (B) and YEF6699-1 [*CDC3-GFP GIC2PBD(W23A)-RFP*] (C) were grown to exponential phase in SC-Ura medium at 25°C and imaged every 2 min for 2 hr at 25°C. Images were generated by average projection of 11 optical slices with 0.6 μm thickness each. Filled triangle indicates the time of bud emergence, which was determined by the first detection of membrane curvature change. Scale bar, 3 μm.

(D and E) The fluorescence intensity of a local cluster of Gic2PBD*-RFP (where * is WT or W23A) was determined as described in Figure S2. Time '0' was defined as the time of bud emergence. The fluorescence intensity of Cdc3-GFP was determined as the total intensity of all pixels in a ROI corresponding to the PBS. Graphs report the average values of 13 and 15 cells, respectively. Error bars represent SEM (Standard Error of the Mean). The values of a Gic2PBD* cluster over time were normalized by the maximum value before bud emergence. For Cdc3-GFP, the values were normalized by the value at the time of bud emergence.

(F) Neither probe affects cell growth or morphology. The wild-type (WT) strains YEF4493-1 (*CDC3-GFP*, left), YZT292-1 (*CDC3-GFP GIC2PBD-RFP*, center), and YEF6699-1 [*CDC3-GFP GIC2PBD(W23A)-RFP*, right] were grown to exponential phase in SC-Leu medium at 37°C and imaged by DIC microscopy.

(G) Cells expressing the improved probe exhibit the same or even lower frequency of chasing than cells expressing no probe at all. The same strains as in (F) were imaged every 2 min for 80 min at 25°C. We counted only the cells that showed recruitment of Cdc3-GFP as a "cloud" at the PBS within the duration of the movie. The cells that showed no movement of Cdc3-GFP cloud from the initial location were categorized as 'no chasing'. The cells that showed movement of Cdc3-GFP signal with a distance shorter than 1 μm (inseparable as distinct ROIs) were categorized as 'slight chasing'. The cells that showed movement of Cdc3-GFP signal with a distance longer than 1 μm (separable as distinct ROIs) were categorized as 'chasing'.

Figure S2. Definition of the Cdc42-GTP Quantification Method, Related to Figure 1

(A) A threshold method was used to determine a local cluster of Cdc42-GTP. The Gic2PBD-RFP signal intensity of each pixel in a cell was measured based on an image

generated by average projection, and the average value and standard deviation were calculated for each cell. If a pixel had intensity higher than the average + 2 standard deviations, the pixel was defined as part of a local Cdc42-GTP cluster. The sum of all pixels selected by this method was used for the analysis.

(B) Validation of the threshold method for detecting a Cdc42-GTP cluster. Cells of a WT haploid strain YEF6699-1 [*CDC3-GFP GIC2PBD(W23A)-RFP*] were grown to exponential phase in SC-Ura medium at 25°C and imaged by dual-color spinning-disk confocal microscopy. Time-lapse image acquisition was performed in every 2 min for 2 hr at 25°C. Shown images were generated by average projection of 11 optical slices with 0.6 μm thickness each. The pixels belonging to the local cluster of Cdc42-GTP are shown in red. The outline of the cell used for analysis is indicated by the yellow dotted line.

Figure S3. Gic1 Binds to Septin Complexes in the Absence of Cdc42-GTP, Related to Figure 2

Septin complexes containing four (4Sep = Cdc3, Cdc10, Cdc11, and His$_6$-Cdc12) or five (5Sep = 4Sep + Shs1) mitotic septins were affinity-purified using Ni-NTA Superflow beads. Purified complexes were incubated with MBP and MBP-Gic1 beads, respectively. After washing, proteins eluted from the beads were resolved by SDS-PAGE and detected by Western blot using anti-MBP (right) and anti-Cdc11 (left) antibodies.

Figure S4. Suppression of Cdc42 Activity by Newly Recruited Septins and Cdc42 GAPs, Related to Figure 3

(A and B) Septins are required for the suppression of Cdc42 activity at the PBS. Cells of a wild-type strain YEF6799-4 [*CDC3-GFP GIC2PBD(W23A)-RFP*] (A) and a septin mutant strain YEF6701-1 [*cdc12-6*, *CDC3-GFP GIC2PBD(W23A)-RFP*] (B) were grown to exponential phase in SC-Ura medium at 25°C and then imaged at 39°C by dual-color spinning-disk confocal microscopy. Before the start of time-lapse imaging, cells were incubated at 39°C for 15 min. Time-lapse image acquisition was performed every 2 min for 2 hr at 39°C. Shown images were generated by average projection of 11 optical slices with 0.6 μm thickness each. Filled triangle indicates the time of bud emergence (A) or the time of initial membrane protrusion (B) that was determined by the first detection of membrane curvature change. Scale bar, 3 μm.

(C, D, and E) GAPs are involved in the negative-feedback regulation of Cdc42 activity. Cells of Cdc42 GAP mutant strains, YEF6667-1 [*bem2*Δ, *CDC3-GFP GIC2PBD(W23A)-RFP*] (C), YEF6700-1 [*rga2*Δ *bem3*Δ, *CDC3-GFP GIC2PBD(W23A)-RFP*] (D), and YEF6692 [*rga1*Δ *rga2*Δ *bem3*Δ, *CDC3-GFP GIC2PBD(W23A)-RFP*] (C) were grown to exponential phase in SC-Ura medium at 25°C and imaged by dual-color spinning-disk confocal microscopy. Time-lapse image acquisition was performed every 2 min for 2 hr at 25°C. Shown images

were generated by average projection of 11 optical slices with 0.7 μm (C) or with 0.6 μm (D, E) thickness each. Filled triangle indicates the time of bud emergence. Scale bar, 3 μm. See also Movie S1.

(F) Bem2-GFP is associated with the nascent septin ring in WT cells at 25°C. Cells of a wild-type strain YEF6948-1 [*BEM2-GFP CDC3-mCherry*] were grown to exponential phase in SC-His medium at 25°C and imaged by dual-color spinning-disk confocal microscopy. Time-lapse image acquisition was performed every 3 min for 2 hr at 25°C. Shown images were generated by maximum projection of 11 optical slices with 0.6 μm thickness each. Yellow triangles indicate septin ring-associated localization of Bem2-GFP. Scale bar, 3 μm.

(G) Bem2 localization at the PBS is decreased in a septin mutant. Cells of a temperature-sensitive septin mutant strain YEF7045-1 [*cdc12-6 BEM2-GFP CDC3-mCherry*] were grown to exponential phase in SC-Leu medium at 25°C and then imaged at 39°C by dual-color spinning-disk confocal microscopy. Before the start of time-lapse imaging, cells were incubated at 39°C for 15 min. Time-lapse image acquisition was performed every 3 min for 2 hr at 39°C. Shown images were generated by maximum projection of 11 optical slices with 0.6 μm thickness each. Each panel shows two selected time points (6 min before bud emergence and 6 min after bud emergence) for a specific cell.

Figure S5. Perturbations of the Plasma Membrane Lipid Composition Do Not Cause Obvious Defects in Septin Ring Assembly and Bud Emergence, Related to Figure 4

(A) Cells of a flippase mutant YEF6908-1 [*lem3Δ CDC3-GFP GIC2PBD(W23A)-RFP*] were grown to exponential phase in SC-Ura medium at 25°C and imaged by dual-color spinning-disk confocal microscopy. Time-lapse image acquisition was performed every 2 min for 2 hr at 25°C. Shown images were generated by average projection of 11 optical slices with 0.6 μm thickness each.

(B) The fluorescence intensity of a local cluster of Gic2PBD(W23A)-RFP was determined as described in Figure S2. Time ‘0’ was defined as the time of bud emergence. The fluorescence intensity of Cdc3-GFP was determined as the total intensity of all pixels in a ROI corresponding to the PBS. Graph reports the average values of 7 cells. Error bars represent SEM (Standard Error of the Mean). The values of a Gic2PBD(W23A) cluster over time were normalized against the maximum value before bud emergence. For Cdc3-GFP, the values were normalized against the value at the time of bud emergence.

(C) Cells of a temperature-sensitive mutant YEF7121-1 [*mss4Δ, (YCplac111mss4-2) (pRS426-GFP-2xPH(PLCδ1))*] defective in PIP2 synthesis and its isogenic wild-type YEF7119-1 [wild-type, (*pRS426-GFP-2xPH(PLCδ1)*)] were grown to exponential phase in SC-Ura medium at 25°C and imaged by spinning-disk confocal microscopy. After setting the sample dish in an environmental chamber at 37°C, image acquisition was started immediately. Time-lapse image acquisition was performed in every 2 min for 1 hr at 37°C. Z-stacks were

11×0.6 μm. Single optical slices are shown.

(D) Ratio of the fluorescence intensity of GFP-2xPH(PLCδ1) (PIP2 probe) at the plasma membrane versus the total cellular signal was quantified as for the cells imaged in (C). Bars indicate average value of 10 cells for each indicated time point after temperature shift. Error bars indicate standard error of the mean.

(E and G) *mss4-2* mutant cells do not display obvious defects in Cdc42 dynamics at the PBS, septin ring assembly, and bud emergence at the restrictive temperature (37°C). Cells of a temperature-sensitive mutant YEF7044-1 [*mss4*Δ *(YCplac111mss4-2) CDC3-GFP GIC2PBD(W23A)-RFP*] (G) and its isogenic wild-type YEF7043-1 [*CDC3-GFP GIC2PBD(W23A)-RFP*] (E) were grown to exponential phase in SC-Ura medium at 25°C and then imaged at 37°C by dual-color spinning-disk confocal microscopy. Before the start of time-lapse imaging, cells were incubated at 37°C for 30 min. Time-lapse image acquisition was performed every 2 min for 2 hr at 37°C. Shown images were generated by average projection of 11 optical slices with 0.6 μm thickness each.

(F and H) The fluorescence intensity of a local cluster of Gic2PBD(W23A)-RFP was determined for the cells imaged in (E) and (G) using the method as described in Figure S2. Time '0' was defined as the time of bud emergence. The fluorescence intensity of Cdc3-GFP was determined as the total intensity of all pixels in a ROI corresponding to the PBS. Graph reports the average values. Error bars represent SEM (Standard Error of the Mean). The values of a Gic2PBD(W23A) cluster over time were normalized against the maximum value before bud emergence. For Cdc3-GFP, the values were normalized against the value at the time of bud emergence.

Figure S6. Effect of *SEC4* Overexpression on Septin Ring Assembly in LatA-treated Cells and Chasing Phenomenon in an Exocytic Mutant, Related to Figure 5

(A and B) Dynamics of Exo84 and septins at the PBS in latA-treated cells with or without *SEC4* overexpression. A WT strain harboring a high-copy-number empty vector [YEF6640-1: *EXO84-GFP CDC3-mCherry* (pRS424)] (A) and a WT strain harboring the vector carrying the *SEC4* gene [YEF6641-1: *EXO84-GFP CDC3-mCherry* (pRS424-*SEC4*)] (B) were grown to exponential phase in SC-Trp medium at 25°C and imaged by dual-color spinning-disk confocal microscopy. Before the start of time-lapse imaging, the cells were pretreated with 100 μM latA for 5 min. Time-lapse image acquisition was performed every 2 min for 80 min at 25°C in the presence of 100 μM latA. Sequence of side-view images of the entire cell (average projection of 11 optical slices with a thickness of 0.6 μm each, top) and sequences of *en face*-view images of a selected region (yellow rectangle) of the chosen cell generated by 3D-reconstruction (bottom) were shown for each strain. Scale bar, 3 μm. See also Movie S5.

(C) Dynamics of Cdc42-GTP and septins during chasing in an exocytic mutant. An exocytic mutant strain YEF6741-1 [*sec4-8*, *CDC3-GFP GIC2PBD(W23A)-RFP*] was grown to exponential phase in SC-Ura medium at 25°C and imaged by dual-color spinning-disk

confocal microscopy. Before the start of time-lapse imaging, the cells were incubated at 37°C for 30 min. Time-lapse image acquisition was performed at 1-min interval for 130 min at 37°C. Shown images were generated by average projection of 11 optical slices with 0.6 μm thickness each. Yellow dotted lines indicate the regions used for quantitative analysis. Scale bar, 3 μm.

(D) Quantification of fluorescence intensity of Gic2PBD-RFP and Cdc3-GFP in the cell shown in (C). Graph reports the sum of the intensities inside each region shown in (C) after background subtraction and normalization.

Supplementary Movie Legends

Movie S1. Dynamics of Cdc42-GTP and Septins during Cell Polarization in Wild-type and Cdc42 GAP Mutant Cells, Related to Figures 1A, 3D, and S4

A wild-type (WT) strain YEF6699-1 and Cdc42 GAP mutant strains YEF6667-1 (*bem2*Δ), YEF6700-1 (*rga2*Δ *bem3*Δ) and YEF6692-1 (*rga1*Δ *rga2*Δ *bem3*Δ) were filmed at 25°C. Scale bar, 3 μm.

Movie S2. Dynamics of Cdc42-GTP and Septins during Cell Polarization in LatA-treated Wild-type and Cdc42 GAP Mutant Cells, Related to Figures 1C and 3F

A wild-type (WT) strain YZT292-1 and a Cdc42 GAP mutant strain YZT393 (*bem2*Δ) were filmed at 25°C in the presence of 100 μM latA. Scale bar, 3 μm.

Movie S3. Simulation of Chasing Phenomenon in the Model, Related to Figure 2

Local concentrations of septins and Cdc42-GTP are color-coded as shades of green and red, respectively.

Movie S4. Formation of a Septin Ring in a Model with Polarized Exocytosis, Related to Figure 4

Local concentrations of septins and Cdc42-GTP are color-coded as shades of green and red, respectively.

Movie S5. Dynamics of Exocyst and Septins during Cell Polarization in Wild-type and Exocytic Mutant Cells and in LatA-treated Wild-Type Cells Harboring an Empty Vector or a *SEC4*-overexpression Plasmid, Related to Figure 5

(0:00) A wild-type (WT) YEF5862-1 and an exocytic mutant YEF6497-1 (*sec4-8*) strains were filmed at 35°C. Scale bar, 3 μm.

(0:21) A wild-type (WT) strain harboring an empty vector (YEF6640-1) and a wild-type strain harboring a *SEC4*-overexpression plasmid (YEF6641-1) were filmed at 25°C in the presence of 100 μM latA. Scale bar, 3 μm.

Supplementary Experimental Procedures

1. Yeast and *E. coli* Strains

Yeast and *E. coli* strains used in this study are listed below. Standard culture media and genetic methods were used in this study (Guthrie and Fink, 1991). Gene deletion was performed using a PCR-based method (Longtine et al., 1998).

Yeast and *E. coli* strains used in this study

Strain name	Org.	Relevant genotype	Source
YEF473A	*S. c.*	**a** *his3 leu2 lys2 trp1 ura3*	(Bi and Pringle, 1996)
YEF473B	*S. c.*	α *his3 leu2 lys2 trp1 ura3*	(Bi and Pringle, 1996)
YEF4493-1	*S. c.*	As YEF473A except *CDC3-GFP:LEU2*	This study
YEF6699-1	*S. c.*	As YEF473A except *GIC2PBD(W23A)-RFP:URA3 CDC3-GFP:LEU2*	This study
YEF6667-1	*S. c.*	As YEF473B except *bem2Δ::KanMX GIC2PBD(W23A)-RFP:URA3 CDC3-GFP:LEU2*	This study
YEF6700-1	*S. c.*	As YEF473A except *rga2Δ::KanMX bem3Δ::TRP1 GIC2PBD(W23A)-RFP:URA3 CDC3-GFP:LEU2*	This study
YEF6692	*S. c.*	As YEF473A except *rga1Δ::HIS3 rga2Δ::KanMX bem3Δ::TRP1 GIC2PBD(W23A)-RFP:URA3 CDC3-GFP:LEU2*	This study
YEF5862-1	*S. c.*	As YEF473A except *EXO84-GFP:URA3 CDC3-mCherry:LEU2*	This study
YEF6497-1	*S. c.*	As YEF473A except *sec4-8 EXO84-GFP:URA3 CDC3-mCherry:LEU2*	This study
YEF6741-1	*S. c.*	As YEF473A except *sec4-8 GIC2PBD(W23A)-RFP:URA3 CDC3-GFP:LEU2*	This study
YEF6640-1	*S. c.*	As YEF473A except *EXO84-GFP:URA3 CDC3-mCherry:LEU2* [pRS424]	This study
YEF6641-1	*S. c.*	As YEF473A except *EXO84-GFP:URA3 CDC3-mCherry:LEU2* [pRS424-*SEC4*]	This study
YZT292-1	*S. c.*	As YEF473A except *GIC2PBD-RFP:URA3 CDC3-GFP:LEU2*	(Tong et al., 2007)
YZT393	*S. c.*	As YEF473A except *bem2Δ::KanMX GIC2PBD-RFP:URA3 CDC3-GFP:LEU2*	This study
YEF743 (M-17)	*S. c.*	**a** *cdc12-6 leu2 ura3*	(Caviston et al., 2003)
YEF6799-4	*S. c.*	As YEF743 except *CDC12 GIC2PBD(W23A)-RFP:URA3 CDC3-GFP:LEU2*	This study
YEF6701-1	*S. c.*	As YEF743 except *cdc12-6 GIC2PBD(W23A)-RFP:URA3 CDC3-GFP:LEU2*	This study
YEF7046-1	*S. c.*	As YEF743 except *CDC12 CDC3-mCherry:LEU2 BEM2-GFP:KanMX*	This study

YEF7045-1	*S. c.*	As YEF743 except *cdc12-6 CDC3-mCherry:LEU2 BEM2-GFP:KanMX*	This study
CCY1024-19C	*S. c.*	α *his3-Δ200 ura3-52 leu2-3,112 trp1-1 gic1-Δ1::LEU2 gic2-1::HIS3*	(Chen et al., 1997)
YEF5805	*S. c.*	As CCY1024-19C except *gic1Δ::KanMX*	This study
YEF5927-2	*S. c.*	As YEF5805 except *CDC3-GFP:LEU2*	This study
YEF5981-1	*S. c.*	As YEF5805 except *CDC3-GFP:LEU2 GIC2PBD-RFP:URA3*	This study
YEF6670-1	*S. c.*	As YEF5805 except *CDC3-GFP:LEU2 GIC2PBD(W23A)-RFP:URA3*	This study
YEF6948-1	*S. c.*	As YEF473A except *BEM2-GFP:HIS3MX6 CDC3-mCherry:LEU2*	This study
YEF6908-1	*S. c.*	As YEF473A except *lem3Δ::TRP1 CDC3-GFP:LEU2 GIC2PBD(W23A)-RFP:URA3*	This study
SEY6210	*S. c.*	α *leu2-3,112 ura3-52 his3-Δ200 trp1-Δ901 suc2-Δ9 lys2-801; GAL*	(Robinson et al., 1988)
YEF7043-1	*S. c.*	As SEY6210 except *GIC2PBD(W23A)-RFP:URA3 CDC3-GFP:TRP1*	This study
YEF7119-1	*S. c.*	As SEY6210 except *[pRS426-GFP-2xPH(PLCδ1)]*	This study
AAY107	*S. c.*	As SEY6210 except *mss4Δ::HIS3MX6 [YCplac111mss4-2]*	(Audhya et al., 2000)
YEF7044-1	*S. c.*	As AAY107 except *mss4Δ::HIS3MX6 [YCplac111mss4-2] GIC2PBD(W23A)-RFP:URA3 CDC3-GFP:TRP1*	This study
YEF7121-1	*S. c.*	As AAY107 except *mss4Δ::HIS3MX6 [YCplac111mss4-2] [pRS426-GFP-2xPH(PLCδ1)]*	This study
BL21(DE3)	*E. c.*	F^- *ompT hsdS* (r_B^- m_B^-) *gal dcm* λ(DE3) (λ(DE3): *lac*I, *lac*UV5-T7 gene 1, *ind*1, *sam*7, *nin*5)	Invitrogen
E1763	*E. c.*	As BL21(DE3) except [pMAL-c2]	This study
E1765	*E. c.*	As BL21(DE3) except [pMAL-c2-*GIC1*]	This study
E1761	*E. c.*	As BL21(DE3) except [pMVB-128-(*CDC10*, His_6-*CDC12*), pMVB133-(*CDC3*, *CDC11*)]	This study
E1897	*E. c.*	As BL21(DE3) except [pMVB-128-(*CDC10*, His_6-*CDC12*), pMVB133-(*CDC3*, *CDC11*), pCOLADuet-(His_6-less)-*SHS1*]	This study

2. Plasmids

All primers were purchased from Integrated DNA Technologies. Plasmid YIp128-CDC3-GFP (integrative, *LEU2*) harbors N-terminally GFP-tagged *CDC3* under the control of its own promoter (Gao et al., 2007). Plasmid YIp128-CDC3-mCherry is the same as YIp128-CDC3-GFP except that the *GFP* ORF (open reading frame) was replaced by the *mCherry* ORF (Gao et al., 2007). YIp128-CDC3-GFP and YIp128-CDC3-mCherry were used for yeast transformation after digestion with *Bgl*II. These sequences are integrated at the *leu2* locus on the genome.

Plasmid YIp211-GIC2PBD-RFP (integrative, *URA3*) carries C-terminally 1.5 copies of tdTomato-tagged *GIC2PBD* (corresponding to the amino acids 1-208 of Gic2) under the control of its own promoter (Tong et al., 2007). To generate plasmid Yip211-GIC2PBD(W23A)-RFP, site-directed mutagenesis was performed by inverse PCR with a pair of primers (GIC2-W23A-3-inv, TTCATCTTCATCCAGCGCAATCGACCGCATCTG and GIC2-W23A-5-inv, GAAGCTGAAAAACTCTACGGTCTCCAGGCCCAG). YIp211-GIC2PBD-RFP and YIp211-GIC2PBD(W23A)-RFP were used for yeast transformation after digestion with *Apa*I. These sequences are integrated at the *ura3* locus on the genome.

Plasmid NRB884 (integrative, *URA3,* containing a 3'-portion of *EXO84* sequence fused in-frame with *GFP* before the stop codon of *EXO84*) (kind gift from Dr. Wei Guo, University of Pennsylvania) was used to generate an *EXO84-GFP* fusion at the endogenous locus under the control of its own promoter. NRB884 was used for yeast transformation after digestion with *Bgl*II.

Plasmid pRS424-SEC4 (2μ, *TRP1*) was constructed by first PCR-amplifying the *SEC4* gene with its own promoter from the template plasmid YGPM-20e05 (Open Biosystems Yeast Genomic Tiling Collection) using a pair of primers (F-SEC4-pRS424, CCCCCCCTCGAGGTCGACGGTATCGATAAGCTTGATATCTTCATATTAGTATAGGATATT and R-SEC4-pRS424, GGCGGCCGCTCTAGAACTAGTGGATCCCCCGGGCTGCAGGGGAAAAAGAATCAAGCGAT), and then gap-repairing the PCR fragment into *Eco*RI-digested pRS424 (Christianson et al., 1992).

3. Development of improved Cdc42-GTP probe

In the course of our experiments, we found that the routinely used Cdc42-GTP probe, *GIC2PBD-RFP*, complemented the temperature-sensitive-growth phenotype of *gic1Δ gic2Δ* cells (Figure S1A). We noted that the same complementation phenomenon was already observed previously (Jaquenoud et al., 1998; Jaquenoud and Peter, 2000). Moreover, a specific mutation (W23A) in *GIC2* was found to eliminate interaction between Gic2 and the

polarisome components and abolished the ability of *GIC2* to complement *gic1Δ gic2Δ* cells without affecting the cellular localization of Gic2. Thus, we introduced the W23A mutation into our Cdc42-GTP probe. This newly constructed probe, *GIC2PBD(W23A)-RFP*, failed to complement *gic1Δ gic2Δ* cells (Figure S1A) as expected but otherwise exhibited the same spatio-temporal dynamics as the old probe (Figure S1 B-E) and exerted no negative effect on cell growth and morphology (Figure S1F). This is in contrast to the full length mutant *GIC2(W23A)* protein that was reported to inhibit budding in a dominant-negative manner when expressed from a galactose-inducible promoter (Jaquenoud and Peter, 2000).

Comparing the two probes we also found that old probe increased the frequency of Cdc42-GTP chasing by septins whereas *GIC2PBD(W23A)-RFP* did not (Figure S1G). Therefore, only strains expressing *GIC2PBD(W23A)-RFP* or no probe were used in this study to quantify the frequency of chasing. This excludes the possibility that the Cdc42-GTP probe affects the probability of chasing reported in this paper.

4. *In Vitro* Protein-Binding Assay

BL21(DE3) *E.coli* strains containing plasmids expressing MBP (pMAL-MBP), MBP-Gic1 (pMAL-Gic1), four septins (Cdc3, Cdc10, Cdc11, His6-Cdc12) [pMVB-128-(CDC10, His_6-CDC12), pMVB133-(CDC3, CDC11)] or five septins (the four septins plus Shs1) [pMVB-128-(CDC10, His_6-CDC12), pMVB133-(CDC3, CDC11), pCOLADuet-NoHisTag-(SHS1)] were induced 3 hours at room temperature with 1 mM IPTG. Cells were washed three times with water, pelleted, and then resuspended in cell lysis buffer (20 mM Tris-HCl, pH 8.0, 300 mM KCl, 5 mM $MgCl_2$, 10 mM β-mercaptoethanol, 0.1% NP-40, 20 mM imidazole (included only for the purification of septin complexes), and EDTA free protease inhibitor (Roche, Cat# 11836170001). Cells were lysed by sonication 6 times (5 amps for 15 sec each time) with an interval of 30 sec on ice in between pulses. Lysate was spun down at 12,000 rpm for 20 minutes and 80% of lysate was collected and spun again at 12,000 rpm for 20 min. The resulting supernatant containing MBP or MBP-Gic1 was added to 500 μL prewashed Amylose resin (BioLabs Cat# E8021L) and incubated for 1 hour at 4°C. After centrifugation and washing with the cell lysis buffer for three times, the Amylose beads bound to MBP or MBP-Gic were used for septin binding reactions. For purification of septin complexes, the supernatant containing the four- or five-septin complexes was added to Ni-NTA Superflow beads (Qiagen Cat# 30410) and incubated for 1 hour at 4°C. After centrifugation and washing the beads with the cell lysis buffer three times, septin complexes were eluted from the beads by adding 500 μL elution buffer (Tris-HCl, pH 8.0, 500 mM KCl, 5 mM $MgCl_2$, 10 mM β-mercaptoethanol, 0.1% NP-40, and 300 mM imidazole) and incubating for 30 min at 4°C. The elution step was repeated three times and the supernatants containing the same septin complexes were pooled together. The septin complexes were concentrated and the cell lysis buffer was changed to the septin-binding buffer (20 mM Tris-HCl, pH 8.0, 75 mM KCl, 5 mM $MgCl_2$, and 10 mM β-mercaptoethanol) using Amicon

Ultra centrifugal filter (Millipore Cat# UFC801024). The MBP and MBP-Gic1 beads were washed three times with the septin-binding buffer and then resuspended in the same buffer. Binding reaction was carried out by mixing 10 μg MBP or MBP-Gic1 with 5 μg septin complexes (four- or five-septin complexes) in a 200 μl reaction volume. The binding reactions were incubated at 4°C for 1 hour. After centrifugation and washing the beads three times with ice-cold septin binding buffer. Proteins associated with the beads were solubilized in 2×SDS-PAGE sample buffer (Santa Cruz Biotechnology Cat# SC-24945). For Western blotting, the primary antibodies were rabbit anti-MBP (NEB Cat# E8030S) and rabbit anti-Cdc11 (y-415) (Santa Cruz Cat # sc-7170) polyclonal antibodies. The secondary antibody was goat anti-Rabbit IgG conjugated to horseradish peroxidase (HRP) (Jackson Immuno-Research Cat # 112-035-175). Signals were detected using chemiluminescent HRP substrate (Millipore Cat# WBKLS0100) on Amersham Hyperfilm ECL (GE Healthcare Cat # 28-9068-39).

5. Live-Cell Imaging

For live-cell imaging, cells were cultured in synthetic complete (SC)-dropout (a specific amino acid or uracil was omitted) media to exponential phase at 25°C. Cells were then embedded in a layer of medium solidified with 1.2% low-melting-temperature agarose (FMC BioProducts) in a poly-lysine coated glass bottom dish (MatTek). When needed, cells were pretreated with 100 μM latA or high temperature (ranging in 35 – 39°C) before the start of live-cell imaging. During time-lapse image acquisition, the temperature of the sample was kept constant in an environmental chamber (Warner Instruments, DH-35). Image acquisitions were performed on a microscope (Olympus, IX71) with a spinning-disk confocal scan head (Yokogawa, CSU10) and a 100× objective lens (Olympus, 1.4NA, Plan S-Apo oil immersion) using an EMCCD camera (Hamamatsu Photonics, ImagEM, C9100-13). Image acquisition was controlled by MetaMorph version 7.7 (Molecular Devices). Two diode lasers (488 nm for GFP and 561 nm for RFP) set in a laser integrator (Spectral Applied Research) were used for excitation illumination. Excitation laser intensities were set in a range from 50% to 80% of the maximal output. An on-chip EM gain setting of 300 was used for the EMCCD camera. Exposure time was set in a range from 40 to 100 msec per acquisition. Z-stacks were in a range from 11×0.6 μm to 11×0.7 μm. Acquisition intervals were set in a range from 1 to 3 min.

For Figure 1A and 1B, cells of a wild-type strain YEF6699-1 [*CDC3-GFP GIC2PBD(W23A)-RFP*] were grown to exponential phase in SC-Ura medium at 25°C and imaged by dual-color spinning-disk confocal microscopy. Time-lapse image acquisition was performed every 2 min for 2 hr at 25°C. Z-stacks were 11×0.6 μm.

For Figure 1C and 1D, cells of a wild-type strain YZT292-1 [*CDC3-GFP GIC2PBD-RFP*]

were grown to exponential phase in SC-Ura medium at 25°C and imaged by dual-color spinning-disk confocal microscopy. Before the start of time-lapse imaging, the cells were pretreated with 100 μM latA for 40 min. Time-lapse image acquisition was performed every 3 min for 150 min in the presence of 100 μM latA at 25°C. Z-stacks were 11×0.6 μm.

For Figure 3A and 3B, cells of a wild-type strain YEF6799-4 [*CDC3-GFP GIC2PBD(W23A)-RFP*] and a septin mutant strain YEF6701-1 [*cdc12-6, CDC3-GFP GIC2PBD(W23A)-RFP*] were grown to exponential phase in SC-Ura medium at 25°C and imaged by dual-color spinning-disk confocal microscopy. Before the start of time-lapse imaging, the cells were treated with a high temperature (39°C) for 15 min. Time-lapse image acquisition was performed every 2 min for 2 hr at 39°C. Z-stacks were 11×0.6 μm.

For Figure 3C and 3D, cells of Cdc42 GAP mutant strains YEF6667-1 [*bem2*Δ, *CDC3-GFP GIC2PBD(W23A)-RFP*], YEF6700-1 [*rga2*Δ *bem3*Δ, *CDC3-GFP GIC2PBD(W23A)-RFP*], and YEF6692 [*rga1*Δ *rga2*Δ *bem3*Δ, *CDC3-GFP GIC2PBD(W23A)-RFP*] were grown to exponential phase in SC-Ura medium at 25°C and imaged by dual-color spinning-disk confocal microscopy. Time-lapse image acquisition was performed every 2 min for 2 hr at 25°C. Z-stacks were 11×0.7 μm (YEF6667-1) or 11×0.6 μm (YEF6700-1 and YEF6692).

For Figure 3E, cells of a wild-type strain YEF7046-1 [*CDC3-mCherry BEM2-GFP*] and a septin mutant strain YEF7045-1 [*cdc12-6, CDC3-mCherry BEM2-GFP*] were grown to exponential phase in SC-Leu medium at 25°C and imaged by dual-color spinning-disk confocal microscopy. Before the start of time-lapse imaging, the cells were treated with a high temperature (39°C) for 15 min. Time-lapse image acquisition was performed in every 3 min for 2 hr at 39°C. Z-stacks were 11×0.6 μm.

For Figure 3F, cells of a Cdc42 GAP mutant strain YZT393 [*bem2*Δ, *CDC3-GFP GIC2PBD-RFP*] were grown to exponential phase in SC-Ura medium at 25°C and imaged by dual-color spinning-disk confocal microscopy. Before the start of time-lapse imaging, the cells were pretreated with 100 μM latA for 40 min. Time-lapse image acquisition was performed in every 3 min for 150 min in the presence of 100 μM latA at 25°C. Z-stacks were 11×0.7 μm.

For Figure 4A and 4B, cells of a wild-type strain YZT292-1 [*CDC3-GFP GIC2PBD-RFP*] were grown to exponential phase in SC-Ura medium at 25°C and imaged by dual-color spinning-disk confocal microscopy. Before the start of time-lapse imaging, the cells were pretreated with 100 μM latA for 40 min. Time-lapse image acquisition was performed every 3 min for 150 min in the presence of 100 μM latA at 25°C. Z-stacks were 11×0.6 μm.

For Figure 5A, 5B, 5C, and 5D, cells of a wild-type strain YEF5862-1 [*EXO84-GFP CDC3-mCherry*] and an exocytic mutant strain YEF6497-1 (*sec4-8 EXO84-GFP CDC3-mCherry*) were grown to exponential phase in SC-Ura medium at 25°C and imaged by dual-color spinning-disk confocal microscopy at 35°C. Before the start of time-lapse imaging, the cells were pretreated with a high temperature of 35°C for 15 min. Time-lapse image acquisition was performed every minute for 1 hr at 35°C. Z-stacks were 11×0.6 μm.

For Figure 5E, Cells of a strain harboring high-copy-number empty vector [YEF6640-1, *EXO84-GFP CDC3-mCherry* (pRS424), wild-type] and a strain harboring a high-copy-number vector containing the *SEC4* gene (*SEC4* O/E, *SEC4* overexpression) [YEF6641-1, *EXO84-GFP CDC3-mCherry* (pRS424-SEC4), wild-type] were grown to exponential phase in SC-Trp medium at 25°C and imaged by dual-color spinning-disk confocal microscopy. Before the start of time-lapse imaging, the cells were pretreated with 100 μM latA for 5 min. Time-lapse image acquisition was performed every 2 min for 80 min at 25°C in the presence of 100 μM latA. Z-stacks were 11×0.6 μm.

For Figure 6A and 6B, cells of a wild-type strain YEF6699-1 [*CDC3-GFP GIC2PBD(W23A)-RFP*] and Cdc42 GAP mutant strains YEF6667-1 [*bem2*Δ, *CDC3-GFP GIC2PBD(W23A)-RFP*], YEF6700-1 [*rga2*Δ *bem3*Δ, *CDC3-GFP GIC2PBD(W23A)-RFP*], and YEF6692 [*rga1*Δ *rga2*Δ *bem3*Δ, *CDC3-GFP GIC2PBD(W23A)-RFP*] were grown to exponential phase in SC-Ura medium at 25°C and imaged by dual-color spinning-disk confocal microscopy. Time-lapse image acquisition was performed every 2 min for 2 hr at 25°C. Z-stacks were 11×0.6 μm (YEF6699-1, YEF6700-1, and YEF6692) or 11×0.7 μm (YEF6667-1).

6. Image Processing and Quantitative Analysis

Image processing and analysis were performed using Fiji (Schindelin et al., 2012). For quantification of fluorescence intensities, image sequences generated by average projection were used. To quantify the intensity of a local cluster of Gic2PBD, a custom-made Fiji macro based on the described below threshold method was used (script available on request).

For Figure 1B, 3A, and 3C, a threshold method was used for quantification of Gic2PBD-RFP (used as a probe for Cdc42-GTP). The Gic2PBD-RFP signal intensity of each pixel in a cell was measured, and after background subtraction, the average value and standard deviation were calculated for each cell. If a pixel had intensity higher than the average + 2 standard deviations, the pixel was defined as belonging to a cluster of Cdc42-GTP. The total intensity of all pixels selected by this method was used in the graphs. The intensities of local clusters of Cdc42-GTP were normalized by the peak value of each cluster before the BE. See also Figure S2.

For Figure 1D, Gic2PBD-RFP signal intensity in each region was calculated as the sum of the pixel intensities in the region after background subtraction. The intensities were normalized by the maximum value in each region.

For quantification of Cdc3-GFP (used as a probe for septins), the sum of the intensities in an ROI corresponding to the PBS after background subtraction was used. The intensities were normalized by the value at BE.

To quantify the septin-ring-opening phenotypes, we analyzed 60-min movies (experiments performed at 35°C) or 80-min movies (experiments performed at 25°C). We counted only the cells that showed Exo84-GFP spot newly formed at the PBS within the duration of the movie.

7. The Model

7.1 Reactions

Complete set of model reactions and notation for the species is presented below. Subsystem of reactions that generate cluster of Cdc42-GTP (RT) (reactions 1-10) has been described earlier (Goryachev and Pokhilko, 2008).

Model reactions and parameters

Common reactions				
#	Reaction	Parameters	Reaction rate	Reference
1	Ec → E	k_1=10 s^{-1}	$v_1=k_1 \cdot Ec$	a
	E → Ec	k_{-1} =10 s^{-1}	$v_{-1}=k_{-1} \cdot E$	
2	E+RD→RT+E	k_2=0.009 $(\mu Ms)^{-1}$	$v_2=k_2 \cdot E \cdot RD$	a
3	RT→RD	k_3=0.0001 s^{-1}	$v_3=k_3 \cdot RT$	a
4	G+RT→RD+G	k_4=0.024 $(\mu Ms)^{-1}$	$v_4=k_4 \cdot G \cdot RT$	b
5	Gc+RT→RD+Gc	k_5=0.024 $(\mu Ms)^{-1}$	$v_5=k_5 \cdot Gc \cdot RT$	b
6	RT+E→M	k_6=10 $(\mu Ms)^{-1}$	$v_6=k_6 \cdot E \cdot RT$	a
	M→ RT+E	k_{-6} =10 s^{-1}	$v_{-6}=k_{-6} \cdot M$	
7	RD+M→RT+M	k_7=0.021 $(\mu Ms)^{-1}$	$v_7=k_7 \cdot M \cdot RD$	a
8	RDIc→RDI	k_8=0.9 s^{-1}	$v_8=k_8 \cdot RDIc$	a
	RDI→RDIc	k_{-8} =0.13 s^{-1}	$v_{-8}=k_{-8} \cdot RDI$	
9	Ic+RD→RDI	k_9=1.5 $(\mu Ms)^{-1}$	$v_9=k_9 \cdot Ic \cdot RD$	a
	RDI→RD+Ic	k_{-9} =0.5 s^{-1}	$v_{-9}=k_{-9} \cdot RDI$	
10	Ec+RT→M	k_{10}=10 $(\mu Ms)^{-1}$	$v_{10}=k_{10} \cdot Ec \cdot RT$	a
11	F→Fc	k_{-11}=0.5 s^{-1}	$v_{-11}=k_{-11} \cdot F$	this study
12	RT+F→RTF	k_{12}=10 $(\mu Ms)^{-1}$	$v_{12}=k_{12} \cdot F \cdot RT$	c, e

	RTF→RT+F	$k_{-12}=6.2\ s^{-1}$	$v_{-12}=k_{-12}\cdot RTF$	
13	RT+Fc→RTF	$k_{13}=1\ (\mu Ms)^{-1}$	$v_{13}=k_{13}\cdot Fc\cdot RT$	this study
	RTF → RT+Fc	$k_{-13}=0.62\ s^{-1}$	$v_{-13}=k_{-13}\cdot RTF$	
14	S→Sc	$k_{-14}=0.02\ s^{-1}$	$v_{-14}=k_{-14}\cdot S$	this study
15	S+S→2P	$k_{15}=0.05\ (\mu Ms)^{-1}$	$v_{15}=k_{15}\cdot S^2$	d
16	S→P	$k_{16}=0.05\ s^{-1}$, $P_{th}=25\mu M$	$v_{16}=k_{16}\cdot \Theta(P-P_{th})\cdot S$	d
17	P→S	$k_{17}=0.1\ s^{-1}$	$v_{17}=k_{17}\cdot P$	this study
18	Gc+P →G+P	$k_{18}=0.05\ (\mu Ms)^{-1}$	$v_{18}=k_{18}\cdot Gc\cdot P$	this study
19	G→Gc	$k_{-19}=0.01\ s^{-1}$	$v_{-19}=k_{-19}\cdot G$	this study
20	Hc+RT→RD+Hc	$k_{20}=0.024\ (\mu Ms)^{-1}$	$v_{20}=k_{20}\cdot Hc\cdot RT$	b
Reactions for hypothesis 1				
21	RTF+Sc→RTFS	$k_{21}=1\ (\mu Ms)^{-1}$	$v_{21}=k_{21}\cdot Sc\cdot RTF$	this study
22	RTF+S→RTFS	$k_{22}=3\ (\mu Ms)^{-1}$	$v_{22}=k_{22}\cdot S\cdot RTF$	this study
	RTFS→RTF+S	$k_{-22}=1\ s^{-1}$	$v_{-22}=k_{-22}\cdot RTFS$	
Reactions for hypothesis 2				
23	FS→FSc	$k_{-23}=2.0\ s^{-1}$	$v_{-23}=k_{-23}\cdot FS$	this study
24	Fc+Sc→FSc	$k_{24}=10\ (\mu Ms)^{-1}$	$v_{24}=k_{24}\cdot Fc\cdot Sc$	this study
	FSc→Fc+Sc	$k_{-24}=0.5\ s^{-1}$	$v_{-24}=k_{-24}\cdot FSc$	this study
25	RT+FSc→RTF+S	$k_{25}=1\ (\mu Ms)^{-1}$	$v_{25}=k_{25}\cdot RT\cdot FSc$	this study
26	F+S→FS	$k_{26}=10\ (\mu Ms)^{-1}$	$v_{26}=k_{26}\cdot F\cdot S$	this study
	FS→F+S	$k_{-26}=0.5\ s^{-1}$	$v_{-26}=k_{-26}\cdot FS$	
27	RT+FS→RTF+S	$k_{27}=1\ (\mu Ms)^{-1}$	$v_{27}=k_{27}\cdot RT\cdot FS$	this study

Total concentrations of species

Species	Concentration [μM]	Number of molecules	Number of molecules from (Ghaemmaghami et al., 2003)
R_{tot} (cdc42)	5	-	-
E_{tot} (cdc24)	0.017	1010	1010
I_{tot} (GDI)	5	-	1670
F_{tot} (GIC)	0.06-0.08	3964	1100 (Gic1), 1130 (Gic2)
S_{tot} (septin)	0.2-0.3	11326	7050 (number of cdc10 /2)
G_{tot} (GAP)	0.005-0.01	566	752 (Bem3), 1230 (Bem2)<br>396 (Rga1), 254 (Rga2)
H_c (GAP)	0.9	-	-

Parameters of diffusion coefficient dependences on septin concentration in Eq. (2)

Species	Diffusion coefficient $D_{i,0}$ $\mu m^2/s$	Diffusion coefficient $D_{i,1}$ $\mu m^2/s$	Constant K_d, μM
E,RT,M,RD,RDI,F,RTF	0.01	$D_{i,0}/50$	60-150
S,RTFS,FS	0.0025	$D_{i,0}/50$	60-150
P,G	0.00025	$D_{i,0}/50$	60-150

Notations: RT, Cdc42-GTP; RD, Cdc42-GDP, E, Cdc24; I, GDI; F, Cdc42 effector; S, septin complex; P, polymerized form of septin; G, Cdc42 GAP recruited by septin, and H_c is a GAP localized only in the cytosol. Symbols without subscript denote concentrations of species on the membrane. Subscript "c" indicates a protein concentration in the cytosol. Concentrations of species correspond to a cell with radius 2.5 μm. $\Theta(P-P_{th})$ is a nucleation step function equal to 1 if $P \geq P_{th}$ and 0 if $P<P_{th}$ where P_{th} is the nucleation threshold for septin polymerization. Reference key: a, (Goryachev and Pokhilko, 2008); b, (Smith et al., 2002); c, (Sudhaharan et al., 2009); d, (Bertin et al., 2010); e, (Hemsath et al., 2005).

We proposed two hypotheses of septin recruitment. According to hypothesis 1 (Figure 2A), cytosolic septin Sc is bound by complex RTF (reaction 21) forming membrane complex RTFS. This complex can diffuse to the periphery of the cluster where the concentration of RT is small. There septin is released from the complex RTFS because its formation on the membrane is reversible (reaction 22).

In hypothesis 2 (Figure 2C), septin can bind effector F forming complex FS and FSc on the membrane (reaction 26) and in the cytosol (reaction 24), respectively. FSc is recruited to the membrane by RT. When FSc is bound to RT it releases S irreversibly (reaction 25).

Further steps of septin accumulation are the same for both hypotheses. Membrane-associated septins may recycle back to the cytosol (reaction 14), or polymerize (reactions 15, 16). Polymer P dissociates back to S via reaction 17.

We assume that Cdc42 GAPs (G) can be reversibly bound by the septin polymer P (reactions 18, 19). Both forms of GAP, the septin-bound (G) and cytosolic (Gc) deactivate RT (reactions 4 and 5, respectively). In our model we also provided for the cytosolic pool of GAP (Hc) that cannot bind septins and deactivates RT through reaction 20.

7.2 Equations

We model yeast cell membrane as a sphere of initial radius R = 2.5 μm. Spatio-temporal dynamics of all variables is defined by the system of linked reaction-diffusion equations

$\frac{\partial c_i}{\partial t} = R_i(\mathbf{c}) + \nabla_A (D_i(\mathbf{c}) \nabla_A c_i)$ where c_i = (E, RT, M, RD, RDI, F, RTF, FS, S, P, G)

(1)

and ∇_A is the gradient on a sphere. The mass-action reaction terms in (1) are as follows:

$R_E = v_1 - v_{-1} - v_6 + v_{-6}$

$R_M = v_6 - v_{-6} + v_{10}$

$R_{RD} = -v_2 + v_3 + v_4 + v_5 - v_7 - v_9 + v_{-9} + v_{20}$

$R_{RDI} = v_8 - v_{-8} + v_9 - v_{-9}$

$R_P = 2v_{15} + v_{16} - v_{17}$

$R_G = v_{18} - v_{19}$

For hypothesis 1

$R_{RT} = v_2 - v_3 - v_4 - v_5 - v_6 + v_{-6} + v_7 - v_{10} - v_{12} + v_{-12} - v_{13} + v_{-13} - v_{20}$

$R_F = -v_{-11} - v_{12} + v_{-12}$

$R_{RTF} = v_{12} - v_{-12} + v_{13} - v_{-13} - v_{21} - v_{22} + v_{-22}$

$R_{RTFS} = v_{21} + v_{22} - v_{-22}$

$R_S = v_{17} - 2v_{15} - v_{16} - v_{-14} - v_{22} + v_{-22}$

For hypothesis 2

$R_{RT} = v_2 - v_3 - v_4 - v_5 - v_6 + v_{-6} + v_7 - v_{10} - v_{12} + v_{-12} - v_{13} + v_{-13} - v_{20} - v_{25} - v_{27}$

R_F=-v_{-11}-v_{12}+v_{-12}-v_{26}+v_{-26}

R_{RTF}=v_{12}-v_{-12}+v_{13}-v_{-13}+v_{25}+v_{27}

R_{FS}=v_{26}-v_{-26}-v_{27}-v_{-23}

R_S=v_{17}-2v_{15}-v_{16}-v_{-14}+v_{25}+v_{27}-v_{26}+v_{-26}

where the elementary reaction rates v_i, v_{-i} and diffusion coefficients are defined in the Supplementary Experimental Procedures, section 7.1.

7.3 Simulation of diffusion

We assume that the lateral diffusion coefficients of membrane-bound species depend on septin polymer concentration in accordance with the commonly accepted notion of the septin diffusion barrier (Caudron and Barral, 2009; Faty et al., 2002; Takizawa et al., 2000). We can consider this dependence using the excluded volume effect approach (Minton, 1989). We assume the simplest exponential dependence for the diffusion coefficient of species on the concentration of P

$$D_i = \left(D_{i,0} - D_{i,1}\right)\exp(-P / K_d) + D_{i,1} \qquad (2)$$

where K_d and $D_{i,1}$=$D_{i,0}$/f are the measures of the strength of the diffusion barrier. We use f = 100 and K_d = 150 μM as realistic assumptions that are comparable, for example, with the dependence of diffusion coefficient of albumin on the concentration of Ficoll 70 (Dix and Verkman, 2008).

Taking into the consideration (2), the diffusion term in equation (1) takes form

$$\nabla(D_i \nabla c_i) = (D_{i,1} - D_i) K_d^{-1} \nabla P \cdot \nabla c_i + D_i \nabla^2 c_i \qquad (3)$$

While the second term in (3) is ordinary Fickean diffusion term, the first term is positive if the gradients of given species *i* and P have opposite signs. Such conditions take place inside the septin ring and the associated term in (3) provides an additional force that focuses the concentration profile of Cdc42-GTP.

7.4 Membrane-cytoplasmic shuttling and mass conservation

We assume that due to the much faster diffusion in the cytosol, cytosolic species can be considered as uniformly distributed. Hence, the equations for the cytosolic species contain only the reaction terms

$$\frac{dc_i}{dt} = R_i(\mathbf{c}), \; c_i = (\mathrm{Ec, Ic, RDIc, Fc, Sc, FSc, Gc}) \qquad (4)$$

Then membrane-cytoplasmic shuttling and cytoplasmic reactions of species can be written as follows:

$$R_{Ec} = \int \left(v_{-1} - v_1 - v_{10}\right) dA / (\eta A)$$

$$R_{Ic} = \int (\mathrm{v}_{-9} - \mathrm{v}_{9}) dA / (\eta A)$$

$$R_{RDIc} = \int (\mathrm{v}_{-8} - \mathrm{v}_{8}) dA / (\eta A)$$

$$R_{Gc} = \int (\mathrm{v}_{19} - \mathrm{v}_{18}) dA / (\eta A)$$

Hypothesis 1

$$R_{Fc} = \int (\mathrm{v}_{-11} - \mathrm{v}_{13} + \mathrm{v}_{-13}) dA / (\eta A)$$

$$R_{Sc} = \int (\mathrm{v}_{-14} - \mathrm{v}_{21}) dA / (\eta A)$$

Hypothesis 2

$$R_{Fc} = \int (\mathrm{v}_{-11} - \mathrm{v}_{13} + \mathrm{v}_{-13}) dA / (\eta A) - \mathrm{v}_{24} + \mathrm{v}_{24}$$

$$R_{Sc} = \int (\mathrm{v}_{-14}) dA / (\eta A) - \mathrm{v}_{24} + \mathrm{v}_{-24}$$

$$R_{FSc} = \int (\mathrm{v}_{-23} - \mathrm{v}_{25}) dA / (\eta A) + \mathrm{v}_{24} - \mathrm{v}_{-24}$$

where integration is taken over the membrane surface, A is the membrane area, reaction rates v_i, v_{-i} are defined in the Supplementary Experimental Procedures, section 7.1 and $\eta = 100$ is the ratio of volumes for the membrane and cytosolic compartments.

Total amount of proteins is conserved within the cell

$$\int (RDI) dA / (\eta A) + RDI_C + I_C = I_{tot} \quad (5)$$

$$\int (E + M) dA / (\eta A) + E_C = E_{tot}$$

Hypothesis 1

$$\int (RT + RD + RDI + M + RTF + RTFS) dA / (\eta A) + RDI_C = R_{tot}$$

$$\int (F + RTF + RTFS) dA / (\eta A) + F_C = F_{tot}$$

$$\int (S + P + RTFS) dA / (\eta A) + S_C = S_{tot}$$

Hypothesis 2

$$\int (RT + RD + RDI + M + RTF) dA/(\eta A) + RDI_C = R_{tot}$$

$$\int (F + RTF + FS) dA/(\eta A) + F_C + FS_C = F_{tot}$$

$$\int (S + P + FS) dA/(\eta A) + S_C + FS_C = S_{tot}$$

$$\int (G) dA/(\eta A) + G_C = G_{tot}$$

Most of simulations in this paper started with all species localized in the cytosol. Initial concentrations on the membrane were

E = M = RT = RDI = RD = F = RTF = FS = S = P = G = RTFS = 0 (6)

while cytoplasmic species were initialized as follows:

$E_c = E_{tot}$, $RDI_c = R_{tot}$, $I_c = 0$, $F_c = F_{tot}$, $S_c = S_{tot}$, $FS_c = 0$ and $G_c = G_{tot}$.

Excluded volume effect needs to be taken into the consideration also in the membrane-cytoplasmic transport terms. Because septin complexes and polymers cover greater area of the membrane than other proteins, we assume the surface-filling effect to depend only on their concentrations. We assume that free area available for the recruitment is a decreasing linear function of the sum of concentrations X = S + P + FS (RTFS in Hypothesis 1) and approaches zero for $X = K_t$. In this case the recruitment of species Z by species Y can be written as

$Z_c + Y \xrightarrow{k} Z + Y$, $k = k'(1 - X/K_t)$ (7)

where k' is the maximal rate. The estimate for K_t may be obtained in a way similar to that for the upper limit of the total concentration of septins. For a sphere with radius R = 2.5 this limit is equal to 1300 μM in the assumption of complete surface coverage.

7.5 Simulation of exocytosis

Following earlier publications (Balaji and Ryan, 2007; Letinic et al., 2009), we consider exocytosis as Poisson process. In addition we assume that its spatially-dependent rate λ is controlled by the concentrations of Cdc42-GTP and septins. Specifically we posit that RT positively influences exocytosis (He and Guo, 2009), while accumulation of septins inhibits it (Amin et al., 2008; Beites et al., 1999). Accordingly, we assume that the local average rate of exocytosis λ increases with RT and decreases with the total concentration of septins on the membrane

$$\lambda(\theta,\varphi) = \lambda_{\exp} \frac{(\lambda_1(\theta,\varphi)+1)dA}{\int (\lambda_1(\theta,\varphi)+1)dA}, \quad \lambda_1(\theta,\varphi) = \frac{RT(\theta,\varphi)^m}{1+X(\theta,\varphi)/K_p} \tag{8}$$

where $\lambda_{\exp}$ is the total experimentally observed intensity of exocytosis, X = P, θ and φ

are angular coordinates on a sphere and the integral is taken over the entire membrane. Because two components of exocyst, Exo70 and Sec3, have been reported to interact with Cdc42-GTP (He and Guo, 2009), we assume that *m* takes value 2.

The value of λ_{exp} corresponds to the rate of cell surface expansion during unpolarized uniform growth and was obtained from the measurements of cell sizes using images produced by our time-lapse live-cell imaging. Assuming the size of exocytic vesicle to be R=0.05 μm (Layton et al., 2011) we found values of λ_{exp} equal to 0.2 s^{-1} and 0.05 s^{-1} for the WT and latA-treated WT cells, respectively.

8. Numerical methods

8.1 Reaction-diffusion equations

Reaction-diffusion equations (1) were solved using explicit finite volume method on a sphere. Making use of spherical system of coordinates (θ_k, φ_j), $k = 1,...,N; j = 1,...,2N$, diffusion term in (1) can be represented as follows:

$$\frac{\Delta c_{i,k,j}}{\Delta t} = \frac{1}{A_{k,j}R}\left[\frac{D_{i,k-1,j} + D_{i,k,j}}{2} ld_{k,j} \frac{c_{i,k-1,j} - c_{i,k,j}}{\Delta\theta_{k-1}} + \frac{D_{i,k+1,j} + D_{i,k,j}}{2} lu_{k,j} \frac{c_{i,k+1,j} - c_{i,k,j}}{\Delta\theta_k} + \right.$$
$$\left. + \frac{D_{i,k,j-1} + D_{i,k,j}}{2} ll_{k,j} \frac{c_{i,k,j-1} - c_{i,k,j}}{\sin\theta_{k+1/2}\Delta\varphi} + \frac{D_{i,k,j+1} + D_{i,k,j}}{2} lr_{k,j} \frac{c_{i,k,j+1} - c_{i,k,j}}{\sin\theta_{k+1/2}\Delta\varphi}\right] \quad (9)$$

where $ld_{k,j}$, $lu_{k,j}$, $lr_{k,j}$ and $ll_{k,j}$ are respectively the lengths of lower, upper, right and left boundaries of the numerical bin (θ_k, φ_j) and $A_{i,j}$ is the bin surface. Poles were treated as single bins (caps). We used a simple explicit algorithm for the diffusion part and super-time-stepping Runge-Kutta algorithm for the time step (Alexiades et al., 1996). The algorithm was validated against analytical solution of the diffusion equation on sphere. Dependence of diffusion coefficients on concentration was computed using Crank approximation for the diffusion flux in which the diffusion coefficient is an algebraic mean of the diffusion coefficients in the neighboring computational bins (Crank, 1975).

8.2 Insertion of exocytic vesicles

Insertion of membrane during exocytosis has been simulated on 1D and planar 2D domains (Layton et al., 2011; Savage et al., 2012). Here we developed a distinct strategy suitable for simulations on a parametrizable closed surface, such as sphere or ellipsoid. We first simulate Poisson process with λ given by (8) using Gillespie next reaction method (Gillespie, 1976). For each time step *t* and numerical bin (θ_k, φ_j) a putative time $\tau_{k,j}$=ln(U_{kj})/ λ_{kj} is computed.

$U_{k,j}$ are independent random numbers uniformly distributed on [0,1] interval. Time of next exocytic event is equal to t + min{$\tau_{k,j}$}. Position of the next exocytic event (θ_{exo},φ_{exo}) is defined by the bin (θ_k, φ_j) that corresponds to min{$\tau_{k,j}$}.

Once the position of exocytic event is chosen, numerical solution is rotated in this way that point (θ_{exo},φ_{exo}) is mapped onto the numerical south pole (π, 0). This re-meshing is obtained by spherical cubic B-spline algorithm (Cotter et al., 2007) which conserves mass. Then, new computational bin of surface equal to that of exocytic vesicle is introduced at the south pole. Boundaries of old bins are recomputed after increasing the sphere radius $R^2_{new}=R^2_{old}+R_v^2$ so that bin surfaces remain constant $A_{i,j}^{new} = A_{i,j}^{old}$. Here R_v is the radius of exocytic vesicle, $A_{i,j}^{new}=R^2_{new}\Delta\varphi[\cos(\theta_{i-1}^{new})-\cos(\theta_i^{new})]$, $A_{i,j}^{old}=R^2_{old}\Delta\varphi[\cos(\theta_{i-1}^{old})-\cos(\theta_i^{old})]$, i=1, …, N and $\theta_0^{old}=\theta_0^{new}=0$. The procedure described above ensures that the profiles of species concentrations are the same outside of new membrane patch before and after the exocytic insertion.

8.3 Simulation of reaction-diffusion on evolving surface

In the exocytosis simulation procedure described above we solve Eqs. (1) on a sphere whose radius slowly increases in time. Once protrusion of bud in the 3rd spatial dimension becomes noticeable, this approach provides only approximate solution.

To analyze the influence of spatial distribution of exocytosis on the cell shape we needed a method capable of simulating reaction-diffusion systems on a moving surface. We used particle-based level set method (Bergdorf et al., 2010) implemented as parallel particle-mesh library client (Sbalzarini et al., 2006). In our simulations evolution of membrane surface A is represented as evolution of set level function ψ moving with velocity **u**

$$\frac{\partial \psi}{\partial t} + \mathbf{u}\nabla\psi = 0 \tag{10}$$

where ψ is a function defined in $\mathbf{R}^3$ which is negative inside, positive outside and equal to zero on A. We assume that the spatial profile of velocity **u** corresponds to the spatial profile of exocytosis as shown in Figure 7C. Concentrations of species are governed by the following advection-diffusion-reaction equation

$$\frac{\partial c_i}{\partial t} + \nabla_A(\mathbf{u}\, c_i) = R_i(\mathbf{c}) + \nabla_A(D_i(\mathbf{c})\nabla_A c_i) \tag{11}$$

where ∇_A is gradient defined on surface A. Initial conditions for particle-based simulations were created by reaction-diffusion system (1) on a sphere of radius R = 2.5μm.

8.4 Simulation of noise

We used Chemical Langevin Equation (CLE) (Gillespie, 2000) approach to introduce internal noise. The CLE is solved by simple 0.5 strong order and weak first order Euler–Maruyama method (Kloeden and Platen, 1992) to solve corresponding stochastic equation for each

computational bin (θ_k, φ_j).

$$c_{i,k,j}(t+\Delta t) - c_{i,k,j}(t) = \sum_{m=1}^{n} n_{i,m} \mathrm{v}_m (c_{k,j}) \Delta t + \sqrt{|n_{i,m}| \mathrm{v}_m (c_{k,j}) \Delta t / (N_A V_{k,j})} dN_m \qquad (12)$$

where N_A is Avogadro number, $V_{k,j}$ is volume of computational bin, dN_m are independent Gaussian random numbers with mean 0 and variance 1, *n* is number of reactions, $v_m(c)$ is the rate of *m*-th reaction shown in the Supplementary Experimental Procedures, section 7.1 and $n_{i,m}$ is stoichiometric coefficient of species *i* in reaction *m*. To avoid heavy computations, we used CLE only for reaction of spontaneous activation of Cdc42, RD → RT with rate constant $k_3 = 10^{-4}\ s^{-1}$ which is approximately equal to the intrinsic nucleotide exchange rate for mammalian Cdc42 (Rossman et al., 2002).

Supplementary References

Alexiades, V., Amiez, G., and Gremaud, P.A. (1996). Super-time-stepping acceleration of explicit schemes for parabolic problems. Communications in Numerical Methods in Engineering *12*, 31-42.

Amin, N.D., Zheng, Y.L., Kesavapany, S., Kanungo, J., Guszczynski, T., Sihag, R.K., Rudrabhatla, P., Albers, W., Grant, P., and Pant, H.C. (2008). Cyclin-dependent kinase 5 phosphorylation of human septin SEPT5 (hCDCrel-1) modulates exocytosis. J Neurosci *28*, 3631-3643.

Audhya, A., Foti, M., and Emr, S.D. (2000). Distinct roles for the yeast phosphatidylinositol 4-kinases, Stt4p and Pik1p, in secretion, cell growth, and organelle membrane dynamics. Mol Biol Cell *11*, 2673-89.

Balaji, J., and Ryan, T.A. (2007). Single-vesicle imaging reveals that synaptic vesicle exocytosis and endocytosis are coupled by a single stochastic mode. Proc Natl Acad Sci U S A *104*, 20576-20581.

Beites, C.L., Xie, H., Bowser, R., and Trimble, W.S. (1999). The septin CDCrel-1 binds syntaxin and inhibits exocytosis. Nat Neurosci *2*, 434-439.

Bergdorf, M., Sbalzarini, I.F., and Koumoutsakos, P. (2010). A Lagrangian particle method for reaction-diffusion systems on deforming surfaces. J Math Biol *61*, 649-663.

Bertin, A., McMurray, M.A., Thai, L., Garcia, G., 3rd, Votin, V., Grob, P., Allyn, T., Thorner, J., and Nogales, E. (2010). Phosphatidylinositol-4,5-bisphosphate promotes budding yeast septin filament assembly and organization. J Mol Biol *404*, 711-731.

Bi, E., and Pringle, J.R. (1996). ZDS1 and ZDS2, genes whose products may regulate Cdc42p in Saccharomyces cerevisiae. Mol. Cell Biol. *16*, 5264-5275.

Caudron, F., and Barral, Y. (2009). Septins and the lateral compartmentalization of eukaryotic membranes. Dev Cell *16*, 493-506.

Caviston, J.P., Longtine, M., Pringle, J.R., and Bi, E. (2003). The role of Cdc42p GTPase-activating proteins in assembly of the septin ring in yeast. Mol Biol Cell *14*, 4051-4066.

Chen, G.-c., Kim, Y.-j., and Chan, C.S.M. (1997). The Cdc42 GTPase-associated proteins Gic1 and Gic2 are required for polarized cell growth in Saccharomyces cerevisiae. Genes

Dev., 2958-2971.

Christianson, T.W., Sikorski, R.S., Dante, M., Shero, J.H., and Hieter, P. (1992). Multifunctional yeast high-copy-number shuttle vectors. Gene *110*, 119-122.

Cotter, C.J., Frank, J., and Reich, S. (2007). The remapped particle-mesh semi-Lagrangian advection scheme. Quarterly Journal of the Royal Meteorological Society *133*, 251-260.

Crank, J. (1975). The mathematics of diffusion (New York: Oxford University Press).

Dix, J.A., and Verkman, A.S. (2008). Crowding effects on diffusion in solutions and cells. Annu Rev Biophys *37*, 247-263.

Faty, M., Fink, M., and Barral, Y. (2002). Septins: a ring to part mother and daughter. Curr Genet *41*, 123-131.

Gao, X.D., Sperber, L.M., Kane, S.A., Tong, Z., Tong, A.H., Boone, C., and Bi, E. (2007). Sequential and distinct roles of the cadherin domain-containing protein Axl2p in cell polarization in yeast cell cycle. Mol Biol Cell *18*, 2542-2560.

Ghaemmaghami, S., Huh, W.K., Bower, K., Howson, R.W., Belle, A., Dephoure, N., O'Shea, E.K., and Weissman, J.S. (2003). Global analysis of protein expression in yeast. Nature *425*, 737-741.

Gillespie, D.T. (1976). General method for numerically simulating stochastic time evolution of coupled chemical-reactions. Journal of Computational Physics *22*, 403-434.

Gillespie, D.T. (2000). The chemical Langevin equation. Journal of Chemical Physics *113*, 297-306.

Goryachev, A.B., and Pokhilko, A.V. (2008). Dynamics of Cdc42 network embodies a Turing-type mechanism of yeast cell polarity. FEBS Lett *582*, 1437-1443.

Guthrie, C., and Fink, G.R. (1991). Guide to yeast genetics and molecular biology. Methods Enzymol. *194*, 933-933.

He, B., and Guo, W. (2009). The exocyst complex in polarized exocytosis. Curr Opin Cell Biol *21*, 537-542.

Hemsath, L., Dvorsky, R., Fiegen, D., Carlier, M.F., and Ahmadian, M.R. (2005). An electrostatic steering mechanism of Cdc42 recognition by Wiskott-Aldrich syndrome proteins. Mol Cell *20*, 313-324.

Jaquenoud, M., Gulli, M.P., Peter, K., and Peter, M. (1998). The Cdc42p effector Gic2p is targeted for ubiquitin-dependent degradation by the SCFGrr1 complex. EMBO J *17*, 5360-5373.

Jaquenoud, M., and Peter, M. (2000). Gic2p may link activated Cdc42p to components involved in actin polarization, including Bni1p and Bud6p (Aip3p). Mol Cell Biol *20*, 6244-6258.

Kloeden, P.E., and Platen, E. (1992). Numerical solution of stochastic differential equations (Berlin: Springer).

Layton, A.T., Savage, N.S., Howell, A.S., Carroll, S.Y., Drubin, D.G., and Lew, D.J. (2011). Modeling vesicle traffic reveals unexpected consequences for Cdc42p-mediated polarity establishment. Curr Biol *21*, 184-194.

Letinic, K., Sebastian, R., Toomre, D., and Rakic, P. (2009). Exocyst is involved in polarized cell migration and cerebral cortical development. Proc Natl Acad Sci U S A *106*, 11342-11347.

Longtine, M.S., Fares, H., and Pringle, J.R. (1998). Role of the yeast Gin4p protein kinase in septin assembly and the relationship between septin assembly and septin function. J Cell Biol *143*, 719-736.

Minton, A.P. (1989). Lateral diffusion of membrane proteins in protein-rich membranes. A simple hard particle model for concentration dependence of the two-dimensional diffusion coefficient. Biophys J *55*, 805-808.

Robinson, J.S., Klionsky, D.J., Banta, L.M., and Emr, S.D. (1988). Protein sorting in Saccharomyces cerevisiae: isolation of mutants defective in the delivery and processing of multiple vacuolar hydrolases. Mol Cell Biol *8*, 4936-4948.

Rossman, K.L., Worthylake, D.K., Snyder, J.T., Cheng, L., Whitehead, I.P., and Sondek, J. (2002). Functional analysis of cdc42 residues required for Guanine nucleotide exchange. J Biol Chem *277*, 50893-50898.

Savage, N.S., Layton, A.T., and Lew, D.J. (2012). Mechanistic mathematical model of polarity in yeast. Mol Biol Cell *23*, 1998-2013.

Sbalzarini, I.F., Walther, J.H., Bergdorf, M., Hieber, S.E., Kotsalis, E.M., and Koumoutsakos, P. (2006). PPM - A highly efficient parallel particle-mesh library for the simulation of continuum systems. Journal of Computational Physics *215*, 566-588.

Schindelin, J., Arganda-Carreras, I., Frise, E., Kaynig, V., Longair, M., Pietzsch, T., Preibisch, S., Rueden, C., Saalfeld, S., Schmid, B., *et al.* (2012). Fiji: an open-source platform for biological-image analysis. Nat Methods *9*, 676-682.

Smith, G.R., Givan, S.A., Cullen, P., and Sprague, G.F., Jr. (2002). GTPase-activating proteins for Cdc42. Eukaryot Cell *1*, 469-480.

Sudhaharan, T., Liu, P., Foo, Y.H., Bu, W., Lim, K.B., Wohland, T., and Ahmed, S. (2009). Determination of in vivo dissociation constant, KD, of Cdc42-effector complexes in live mammalian cells using single wavelength fluorescence cross-correlation spectroscopy. J Biol Chem *284*, 13602-13609.

Takizawa, P.A., DeRisi, J.L., Wilhelm, J.E., and Vale, R.D. (2000). Plasma membrane compartmentalization in yeast by messenger RNA transport and a septin diffusion barrier. Science *290*, 341-344.

Tong, Z., Gao, X.-D., Howell, A.S., Bose, I., Lew, D.J., and Bi, E. (2007). Adjacent positioning of cellular structures enabled by a Cdc42 GTPase-activating protein–mediated zone of inhibition. J. Cell Biol. *179*, 1375-1384.
