# Supplementary figures and images for: Daughter Cell Identity Emerges from the Interplay of Cdc42, Septins, and Exocytosis
Source: Dev Cell. 2013 Jul 29;26(2):148–61. doi: 10.1016/j.devcel.2013.06.015 (PMC3730058; doi:10.1016/j.devcel.2013.06.015)

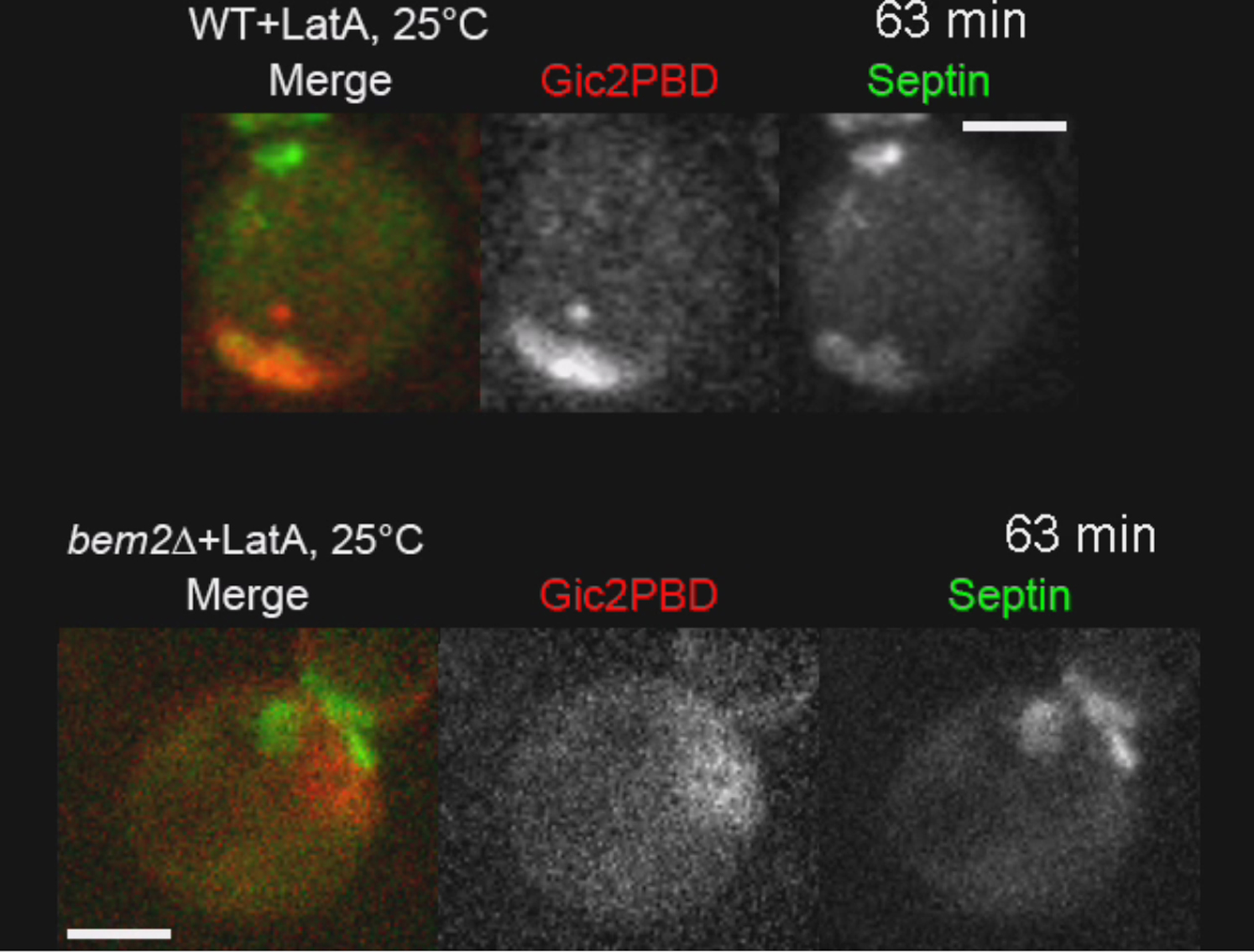

Supplement: Movie S2. Dynamics of Cdc42-GTP and Septins during Cell Polarization in LatA-Treated Wild-Type and Cdc42 GAP Mutant Cells, Related to Figures 1C and 3F — A WT strain YZT292-1 and a Cdc42 GAP mutant strain YZT393 (bem2Δ) were filmed at 25°C in the presence of 100 μM latA. Scale bar, 3 μm. [file mmc3.jpg]

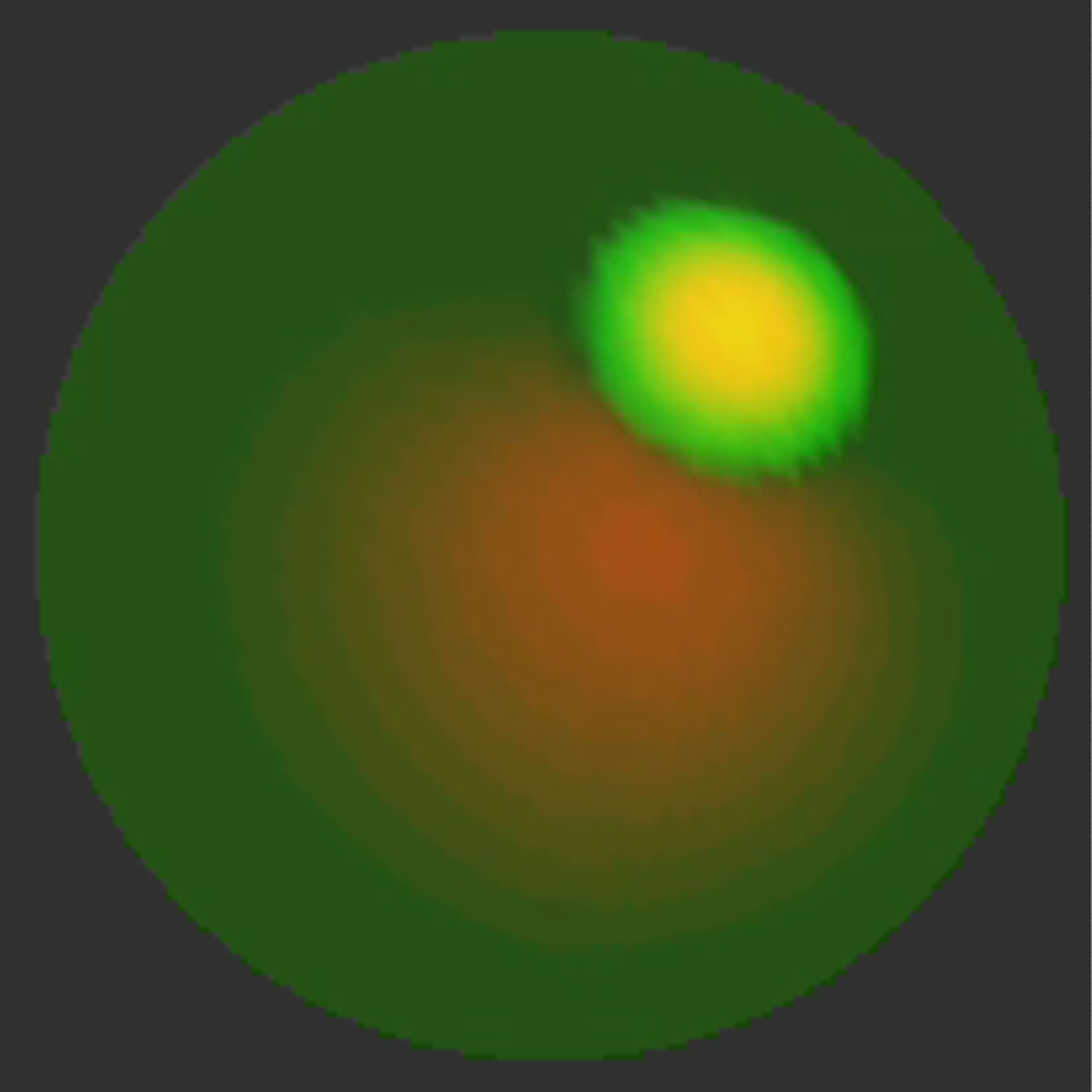

Supplement: Movie S3. Simulation of Chasing Phenomenon in the Model, Related to Figure 2 — Local concentrations of septins and Cdc42-GTP are color-coded as shades of green and red, respectively. [file mmc4.jpg]

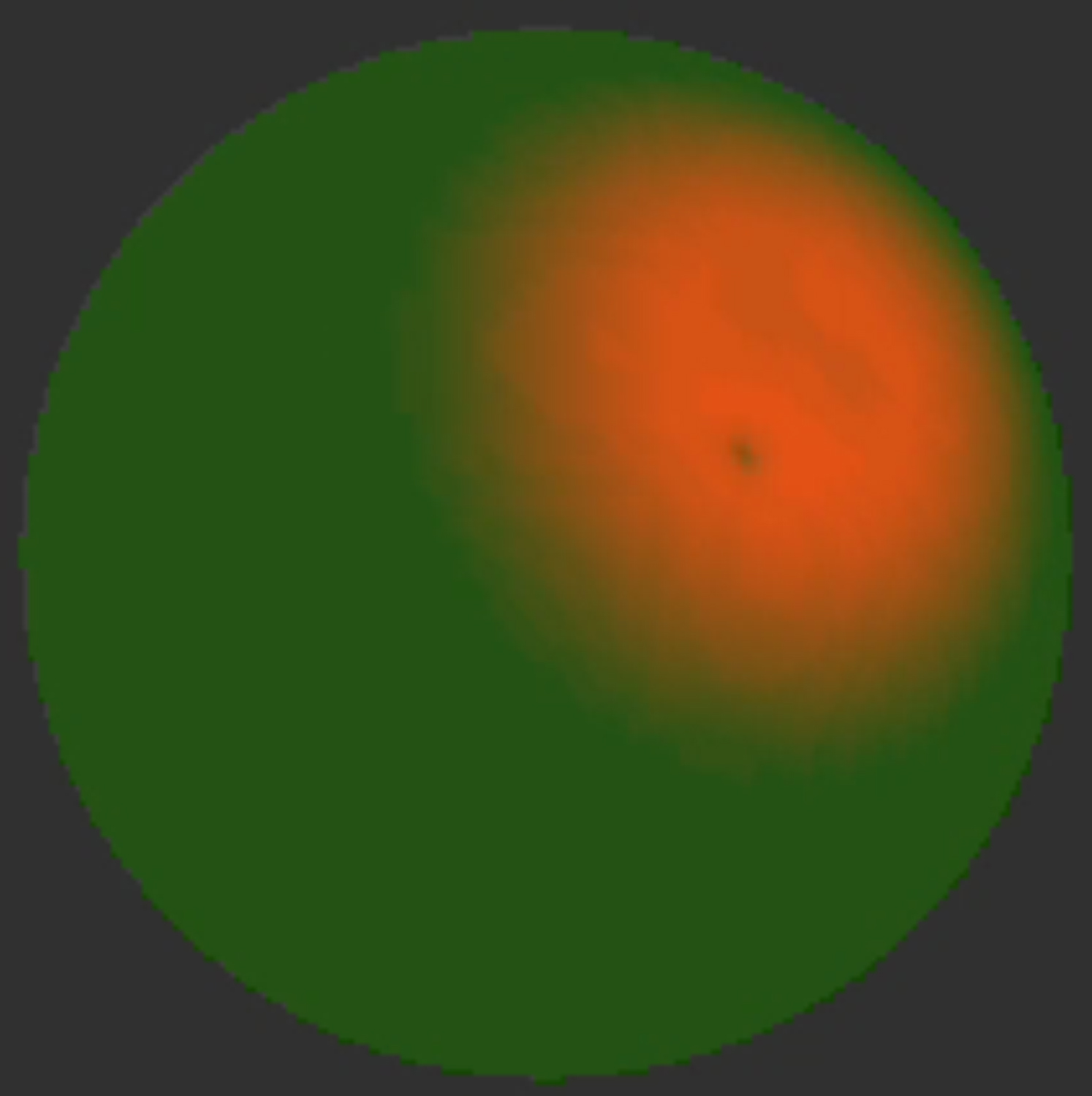

Supplement: Movie S4. Formation of a Septin Ring in a Model with Polarized Exocytosis, Related to Figure 4 — Local concentrations of septins and Cdc42-GTP are color-coded as shades of green and red, respectively. [file mmc5.jpg]

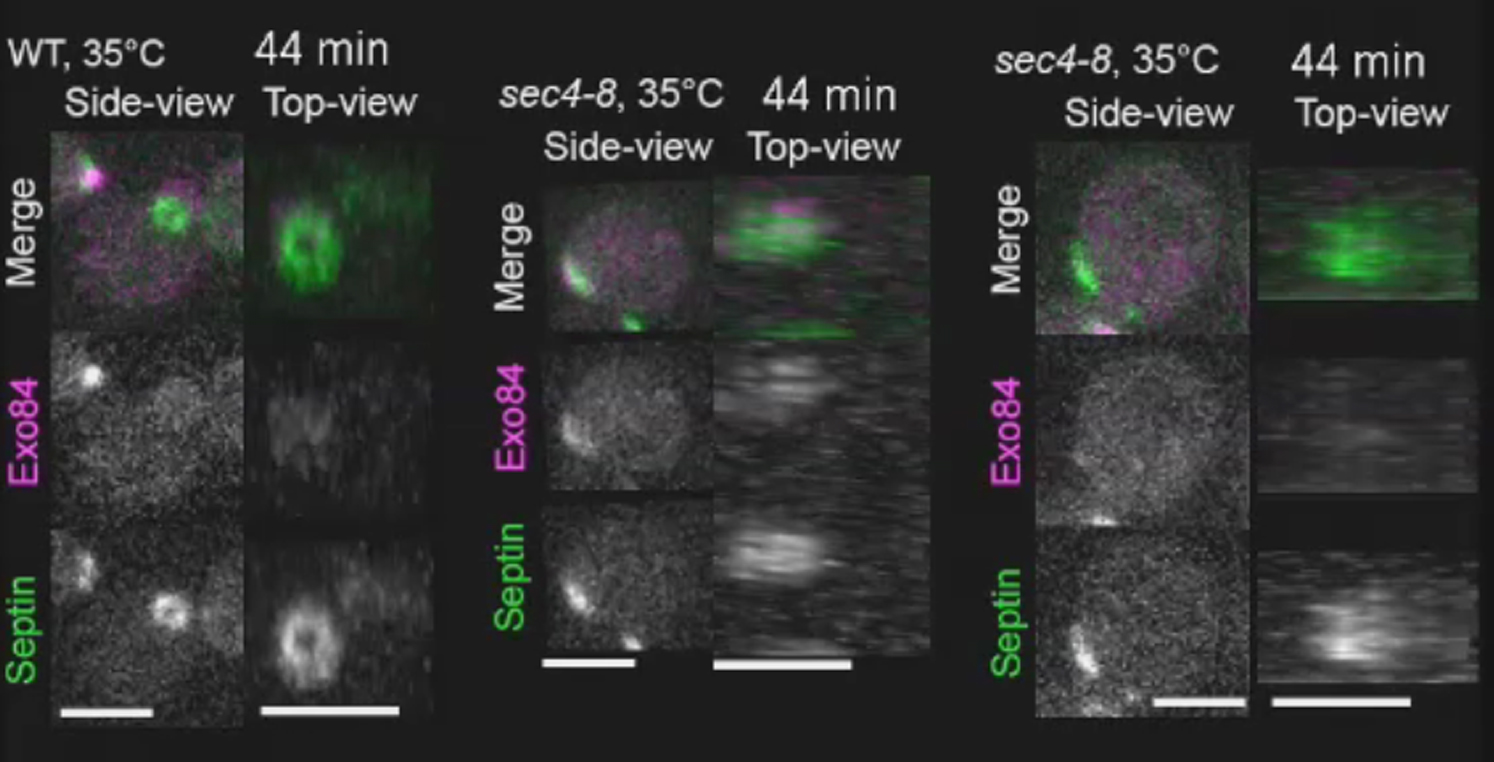

Supplement: Movie S5. Dynamics of Exocyst and Septins during Cell Polarization in Wild-Type and Exocytic Mutant Cells and in LatA-Treated Wild-Type Cells Harboring an Empty Vector or a SEC4-Overexpression Plasmid, Related to Figure 5 — (0:00) A WT YEF5862-1 and an exocytic mutant YEF6497-1 (sec4-8) strain were filmed at 35°C. Scale bar, 3 μm. (0:21) A WT strain harboring an empty vector (YEF6640-1) and a wild-type strain harboring a SEC4-overexpression plasmid (YEF6641-1) were filmed at 25°C in the presence of 100 μM latA. Scale bar, 3 μm. [file mmc6.jpg]
